# Pharmacophore-based discovery of 2-(phenylamino)aceto-hydrazides as potent eosinophil peroxidase (EPO) inhibitors

**DOI:** 10.1080/14756366.2018.1512598

**Published:** 2018-10-04

**Authors:** Daniela Schuster, Martina Zederbauer, Thierry Langer, Andreas Kubin, Paul G. Furtmüller

**Affiliations:** a Institute of Pharmacy, Division of Pharmaceutical and Medicinal Chemistry, Paracelsus Medical Private University Salzburg, Salzburg, Austria;; b Planta Naturstoffe, Vienna, Austria;; c Department of Pharmaceutical Chemistry, Division of Drug Design and Medicinal Chemistry, Faculty of Life Sciences, University of Vienna, Vienna, Austria;; d Department of Chemistry, Division of Biochemistry, University of Natural Resources and Life Sciences, Vienna, Austria

**Keywords:** Eosinophil peroxidase inhibitors, 2-(phenylamino)aceto-hydrazides derivatives, mechanism of inhibition, structure-activity-relationship, pre-steady-state kinetics

## Abstract

There is an increasing interest in developing novel eosinophil peroxidase (EPO) inhibitors, in order to provide new treatment strategies against chronic inflammatory and neurodegenerative diseases caused by eosinophilic disorder. Within this study, a ligand-based pharmacophore model for EPO inhibitors was generated and used for *in silico* screening of large 3 D molecular structure databases, containing more than 4 million compounds. Hits obtained were clustered and a total of 277 compounds were selected for biological assessment. A class of 2-(phenyl)amino-aceto-hydrazides with different substitution pattern on the aromatic ring was found to contain the most potent EPO inhibitors, exhibiting IC_50_ values down to 10 nM. The generated pharmacophore model therefore, represents a valuable tool for the selection of compounds for biological testing. The compounds identified as potent EPO inhibitors will serve to initiate a hit to lead and lead optimisation program for the development of new therapeutics against eosinophilic disorders.

## Introduction

Eosinophil peroxidase (EPO), a member of the peroxidase-cyclooxygenase superfamily^1^ is a fundamental component of the leukocytes granule – the eosinophils that are specialised human phagocytes with critical role in eliminating tissue-invasive large parasites. Beside its beneficial contribution in the innate immunity although its role in pathogenesis of eosinophilic disorders has been discussed^2–4^. There is clear evidence that EPO is the main toxic protein of the eosinophil granule proteins. In the case of eosinophilic disorders and accumulation of eosinophils in various organs EPO contributes to chronic inflammatory^5–8^ and neurodegenerative diseases^9^.

Eosinophils are produced in bone marrow and normally lie within the range of 0.5–4% of total white blood cells (∼30–300 cells per µL)^10^. Increased concentration in blood can be observed in the case of infectious diseases, allergic reactions and inflammation and destructive capabilities^11^. Eosinophils penetrate through the vascular wall and accumulate in the affected tissue and body fluids. Most eosinophils are found in the connective tissue immediately beneath the epithelium of the gut, urogenital and respiratory tract. At this location they release diverse biologically active molecules, like EPO, cytokines, chemokines and membrane-derived mediators^12^. The molecules stored in the eosinophils are released into the tissue through degranulation in order to inactivate attacking microbes and parasites for example, worms such as Helminths^13^ and Schistosomas^14^, as well as tissue invading parasites^15^. Eosinophils are also activated in the presence of allergens, particularly in the respiratory tract^16^. Patients with allergic asthma and allergic rhinitis show comparable local accumulation of eosinophils, comparable degranulation patterns, which causes chronic inflammation in the pathogenesis of respiratory diseases^16^. Very recently it was shown that EPO-generated oxidants mediated mucus plugs formation in patients with asthma linked to eosinophilia and airflow obstruction^17^. In their proposed model thiocyanate is oxidised by EPO to hypothiocyanite that targets cysteine thiols groups in the secreted mucin polymer to generate covalent disulfide mucin crosslinks. Crosslinked mucins have a high elasticity that decreases their clearance by the mucocilliary escalator and results in mucus plug formation. In another study, it was shown that EPO-mediated protein carbamylation is promoted during allergen-induced asthma exacerbation, and can both modulate immune responses and trigger a cascade of many of the inflammatory signals present in asthma^18^. Eosinophils can accumulate in regions where body’s own tissue or cells are misguided and respectively shows malfunction: in the case of endometriosis, eosinophils, primarily the main granule protein EPO is suspected of contributing to the chronic inflammatory processes of endometriosis^19^. The role of eosinophils in the pathogenesis of Parkinson’s disease has also been discussed. Eosinophil accumulation and intracytoplasmatic eosinophilic inclusions (Lewy bodies) in neurons of the substantia nigra and other brain regions are observable^9^. Further evidence for the contribution of eosinophils in neurodegenerative diseases particularly in patients suffering from amyotrophic lateral sclerosis (ALS) is given by Liu et al.^20^.

If the number of eosinophils in tissue or blood is significantly high and persistent then the so-called eosinophilia becomes manifest and chronic inflammatory processes and organ damage are observable^12^. If degranulation and the release of EPO and the granule proteins do not occur, chronic inflammation processes are not initiated^21^. Therefore, in the case of a local accumulation of eosinophils, degranulation is considered a key pathogenic event in major chronic eosinophilic diseases^22^, also called eosinophilia and eosinophilic disorder respectively.

EPO is the protein with the highest mass fraction within the cationic proteins, released out of eosinophils with over 40% of the granule protein mass^23^. It displays a direct toxic effect, and is held mainly responsible for inflammation and organ damage. Parra et al^24^ described the tissue-damaging consequences of EPO which are relative to EPO concentration and the progress of the chronic development of eosinophilic disorders. In the case of bronchial asthma EPO concentration in the plasma of healthy test persons is ∼10 ng/mL, whereas the plasma of asthma patients reveals 40 ng/mL EPO – four times higher than normal values. In the case of bronchial asthma EPO concentration is evenly balanced in proportion to the progress and stages of asthma^4^.

EPO knockout mice in a dextran sulfate-induced model of ulcerative colitis provide clear evidence of the essential role of EPO in the pathogenesis of colitis^25^. In this model there was distinct evidence of eosinophil degranulation and the release of EPO into the colon lumen. The impact of EPO in pathogenesis was confirmed by the findings that EPO knockout mice had significantly less weight loss, colon shrinkage, and colonic bleeding than their wild-type counterparts. Moreover, treatment with the potent EPO inhibitor resorcinol similarly diminished these three disease activity parameters in wild-type mice. Interestingly, a major basic protein knockout mouse line showed no change in pathology in comparison with wild-type animals^25^.

Our stated goal is to develop potent inhibitors against EPO in order to provide new treatment strategies against chronic inflammatory and neurodegenerative diseases caused by eosinophilic disorder. In order to discover novel EPO inhibitors, a pharmacophore model was developed and used for *in silico* screening of millions of compounds from commercially available sources and subsequently selecting substances for biological testing. Active virtual hits were further investigated by testing structurally related compounds for their EPO inhibitory activity and establish structure-activity-relationship (SAR) rules. This study finally provides a series of EPO-inhibiting 2-(phenyl)amino-aceto-hydrazides^26^ as candidates for further biological investigations and lead optimisation.

## Experimental section

### Pharmacophore model

All 3 D structures and their conformations were calculated within Accelrys Catalyst version 4.11 (San Diego, CA, USA). For the generation of 3 D multi-conformational compound databases of the training set and test set molecules, BEST conformational calculations were employed with a maximum of 250 conformations per molecule and an energy maximum of 20 kcal above the calculated energy minimum. The 3 D multi-conformational structure databases of commercially available compounds were calculated using the FAST settings with max. 50 conformers per molecule. Pharmacophore models were calculated within Accelrys Catalyst version 4.11 using the HipHop common feature model algorithm. Screening of the training and test set database was done using the BEST FLEXIBLE search algorithm, which allows the compounds to optimise their conformations during the fitting procedure, so that they geometrically better map the pharmacophore features. Filtering of the hit lists using Lipinski rules and structural clustering were performed using the Lipinski filtering protocol and the chemical diversity clustering protocol of Pipeline Pilot. For the chemical clustering, ECFP_6 was used with a maximum cluster distance of 0.7 and 50 clusters. For the SAR studies, structurally related, commercially available compounds were searched using SciFinder. Only compounds with a minimum Tanimoto coefficient of 0.8 compared to the original hits were considered.

### Eosinophil peroxidase and chemicals

Eosinophil peroxidase was purified from human white blood cells to a purity index (*A*_413_/*A*_280_) of at least 1.0 as described by Olsen and Little^27^. Its concentration was calculated using *ε*_413 nm_ 110 000 M^−1 ^cm^−128^ Hydrogen peroxide, obtained as a 30% solution from Sigma Chemical Co., was diluted and the concentration determined by the absorbance measurement at 240 nm where the extinction coefficient is 39.4 M^−1 ^cm^−129^. The other chemicals were also purchased from Sigma Chemical Co. at the highest grade available.

### Steady-state experiments

Halogenation activity was measured spectrophotometrically (Hitachi U-3000) using the monochlorodimedone (MCD) assay^30^. MCD (100 µM) was dissolved in 100 mM phosphate buffer, pH 7.0 containing either bromide (100 mM) or chloride (100 mM) and 20 nM EPO. Upon addition of 100 µM H_2_O_2_ MCD was converted into dibromodimedone. Rates of halogenation were determined from the initial linear decrease of the time traces using an extinction coefficient for MCD at 290 nm of 19.9 mM^−1 ^cm^−1^. If necessary the inhibitor stock solutions were prepared in dimethylsulfoxide (DMSO) and stored in dark flasks. Dilution was performed with 100 mM phosphate buffer, pH 7.0, to a final DMSO concentration of maximal 2% (v/v) in all assays. Concentrations up to 10% (v/v) DMSO showed no influence on the bromiation activity of eosinophil peroxidase. The inhibitor concentration that inhibited eosinophil peroxidase-dependent oxidation of MCD by 50% (IC50) was determined by fitting a rectangular hyperbola to the dose-response curve using nonlinear regression (see supplemental information A). All measurements were made in triplicate.

### Pre-steady-state experiments

The sequential stopped-flow apparatus (model SX-18 MV) and the associated computer system were from Applied Photophysics (UK). Because of the inherent instability of Compound I of EPO, the sequential stopped-flow (multi-mixing) technique was used for determination of rate of the reaction of Compound I with different inhibitors. Similar to bovine LPO, human EPO Compound I could be formed with equimolar concentrations of H_2_O_2_. In a typical experiment, 2 µM EPO was premixed with 2 µM H_2_O_2_ in the aging loop. After 100 ms of delay, the formed Compound I was mixed with increasing concentrations of different inhibitors. Formation and reduction of Compound II by compound **10** was investigated by following the biphasic time trace at 423 nm, where the transition of Compound I to Compound II and further to the Compound III can be followed. The kinetic traces were fitted using the double-exponential equation of the Applied Photophysics software. At least three determinations (2000 data points) of pseudo-first-order rate constants (*k*_obs_) from the first phase of the reaction were performed for each substrate concentration and the mean value was used in the calculation of the second-order rate constants, which were calculated from the slope of the line defined by a plot of *k*_obs_ versus substrate concentration. Compound **11** was investigated by following the biphasic time traces at 430 nm the maximum absorbance of Compound II. All reactions were followed both at single wavelengths as well as with a diode-array detector. Polychromatic data were analysed with Pro-Kineticist from Applied Photophysics. The program simultaneously fits the kinetic traces at all wavelengths to the proposed reaction mechanism and simulates the spectra of all reactant, product and intermediate species as well as their time-dependent distribution on the reaction coordinate. All measurements were done in 100 mM phosphate buffer, pH 7.0, at 25 °C with a minimum of three repeats.

## Results and discussion

**Figure 1.**
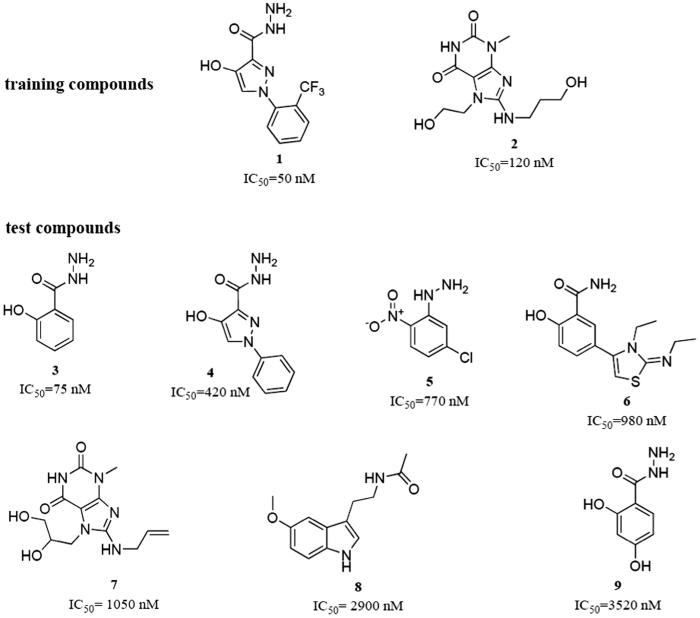
Training and test set compounds used for the generation and theoretical validation of the EPO inhibitor pharmacophore model.

In a first step due to the absence of an X-ray structure of EPO an exhaustive 3 D model of EPO was generated using the structure of lactoperoxidase (2R5L) as templet. The structure of LPO was chosen because LPO shows the highest sequence homology to EPO and both proteins have the same active site architecture with posttranslationally modified heme *b* as cofactor. In the second step, pharmacophore models, featuring the 3 D key chemical determinants required for binding to EPO, were generated using the structural data of nine potent myeloperoxidase inhibitory compounds known so far as modeling dataset (Figure 1).

The model was generated based on the compounds **1** and **2**. These two training molecules were selected because of their high activity and structural dissimilarity. Due to the structural features of the training compounds, the program was allowed to use hydrogen bond acceptors (HBAs), hydrogen bond donors (HBDs), hydrophobic (Hy), aromatic hydrophobic (HyAr), aromatic rings (AR), and positively ionisable (PI) pharmacophore features for the model generation. Ten models were obtained from the model generation process. All of them contained six pharmacophore features. The models were quite similar among each other. They mainly differed in two aspects: Some Hy features were replaced by AR features and HBAs were exchanged with HBDs, or *vice versa*.

For selecting the most suitable model for finding putative EPO inhibitors from large chemical databases, three criteria were set: (i) good fitting of the training compounds to the model with a preferably high geometric fit value, and (ii) good retrieval of the test compounds (Figure 1) from the data set. (iii) Because we were most interested in small molecules as hits, only fitting compounds with a molecular weight <300 g/mol were considered. All ten models were evaluated against these criteria. The results of these experiments are shown in Table 1.

**Table 1.** The ten generated EPO inhibitor pharmacophore models with the geometric fit values of the training and test compounds. The model selected for further virtual screening purposes is highlighted in green.

Cpd.	EPO IC_50_ [nM]	Model 1	Model 2	Model 3	Model 4	Model 5	Model 6	Model 7	Model 8	Model 9	Model 10
Trainingset											
1	50	3.38	3.40	1.73	3.42	3.42	3.55	1.76	1.78	3.94	3.89
2	120	6.00	6.00	6.00	6.00	6.00	6.00	6.00	6.00	6.00	6.00
Testset											
3	75	1.17	1.27	n.f.^a^	1.52	1.44	1.66	n.f.	n.f.	1.40	1.22
4	420	2.05	2.32	1.88	2.51	2.49	2.54	2.22	2.14	3.24	3.21
5	770	n.f.	n.f.	n.f.	n.f.	n.f.	n.f.	n.f.	n.f.	2.55	1.99
6	980	n.f.	n.f.	n.f.	n.f.	n.f.	n.f.	n.f.	n.f.	1.75	2.27
7	1050	5.20	5.25	5.01	5.66	5.39	5.62	5.56	5.57	5.09	5.47
8	2900	n.f.	n.f.	n.f.	n.f.	n.f.	n.f.	n.f.	n.f.	0.76	1.18
9	3520	1.02	1.13	n.f.	1.37	1.29	1.52	0.06	n.f.	1.22	1.03

a n.f.: not fitting.

**Figure 2.**
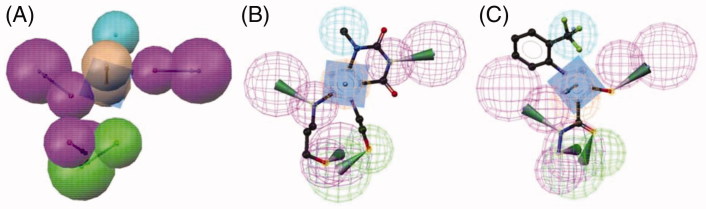
(A) EPO inhibitor model 5 composed out of six chemical features: three HBDs (magenta), one HBA (green), one Hy (cyan), and one AR (brown). The training compounds **1** (B) and **2** (C) fitted into the pharmacophore are shown.

Based on the results presented in Table 1, model 5 (Figure 2) was selected for the further screening experiments. Model 5 was preferred over models 9 and 10, which were able to correctly retrieve all training and test set compounds, because of its higher restrictivity in screening test databases of drug-like compounds. In this cherry picking scenario, it is more important to find the majority of active compounds in a preferably small hit list than to find all active compounds.

Model 5 was used for exhaustive database mining. In total, 17 commercially available compound collections were collected, transformed into searchable 3 D multi-conformational databases, and virtually screened using model 5. As mentioned above, only hits with a maximum molecular weight of 300 were further processed (Table 2).

**Table 2.** Hits with a maximum molecular weight of 300 g/mol for further processing.

Database	DB^a^ size	DB size with MW <300	no. hits	% DB <300	% whole DB
Asinex Gold	227864	54470	525	0.96	0.23
Asinex Platinum	130352	7343	61	0.83	0.05
Chemblock	123345	24288	237	0.98	0.19
Chembridge	455529	108280	512	0.47	0.11
Chemdiv DC	682329	66980	583	0.87	0.09
Chemdiv NC	22349	1547	4	0.26	0.02
Enamine	495601	65943	181	0.27	0.03
Interbioscreen NC	42222	6151	114	1.85	0.27
Interbioscreen SC	373985	47252	557	1.18	0.15
Lifechem STK	296076	31004	298	0.96	0.10
Lifechem TAN	349377	9949	39	0.39	0.01
Maybridge	56842	21000	119	2.10	0.77
NCI	247041	129422	1224	0.95	0.50
Specs NC	323	75	1	1.33	0.31
Specs SC	199165	35283	154	0.43	0.08
Vitas M Stock	316410	51967	362	0.70	0.11
Vitas M Tulip	24670	2669	57	2.14	0.23
Total	4043480	663623	5028	0.76	0.12

a DB: database.

In total, over 660 000 small molecular weight (<300 g/mol) out of over 4 million database compounds were virtually screened, leading to a preliminary hit list of 5028 structures. After the removal of duplicates, 2401 unique compounds remained. The compounds were then filtered according to Lipinski rules (2364 compounds passing) and clustered by chemical diversity (ECFP_6, Max Distance 0.7). In total, this process returned 291 clusters. For each cluster, the compound with the highest BestFit value was highlighted as preferable test compound. All test candidates were checked for possible substructures that are typical for pan assay interference compounds (PAINS: http://zinc15.docking.org/patterns/home/)^31^. According to this substructure filter, no suspected PAINS were present among the selected test compounds.

### Steady-state kinetics

The 277 selected compounds were tested for inhibition. Monochlorodimedon (MCD) was used to monitor inhibition of EPO-mediated bromination^30^. It turned out that certain compounds such as hydrazides are able to inhibit the EPO. Table 3 lists the IC_50_ values of four different 4-chlorophenyl derivatives of hydrazides and acetic acid. The presented IC_50_ values obviously revealed that the capacity of the investigated compounds to inhibit EPO-mediated bromination was modulated by the functional groups in position 2. 2-aminoaceto-hydrazide compounds are among the most active compounds with the highest inhibition capacity followed by acetic acid, whereas 2-thioaceto-hydrazide or (2-phenoxy)aceto-hydrazide showed much less inhibition. It is apparent that a free terminal amino group, which could act as an electron acceptor is essential for a strong inhibition, additional to steric and/or electrochemical properties of these compounds.

**Table 3.** IC_50_ of EPO inhibitors in dependence of substituents of functional groups in position 2

Compound	Structure	IC_50_ value (µM)
**10** (2-[(4-chlorophenyl) amino]- acetohydrazide; PAAH)		0.021 ± 0.005
**11** (2-[(4-chlorophenyl) amino]- acetic acid; PAAA)		0.205 ± 0.03
**12** (2-(4-chlorophenyl) thio-acetohydrazide)		1.200 ± 0.08
**13** (2-(4-chlorophenoxy)-aceto-hydrazide)		4.900 ± 0.1

In order to obtain more detailed information, we analysed the dependence of the position and the substituents on benzene ring (Table 4). From the analysis of the IC_50_ values it becomes clear that substituents in position 4 play an important role. The highest inhibitory activity can be found if chlorine is in this position, followed by fluorine and iodine, whereas bromine shows the lowest activity. If position 4 is free, or there is a methyl group, the amino-aceto-hydrazide shows very weak inhibitory effect compared to the halides substituents. The lowest IC_50_ values can be found when fluorine is in position 2 or if two fluorine atoms occupy position 2 and 4. A similar IC_50_ value was measured with the mixed halide, when chlorine was in position 3 and fluorine in position 4. Less important positions are position 6 and position 3, if they are occupied with one halide.

**Table 4.** IC_50_ of EPO inhibitors in dependence of substituents on benzene ring.

Compound	Substituents	IC_50_ value
		(µM)
**14** (PAAH)	–	2.900 ± 0.1
**15** (4-Methyl-PAAH)	4-CH_3_	2.270 ± 0.09
**16** (4-Fluoro-PAAH)	4-F	0.172 ± 0.03
**17** (4-Bromo-PAAH)	4-Br	0.823 ± 0.08
**18** 4-Iodo-PAAH	4-I	0.290 ± 0.02
**19** 2-Fluoro-PAAH	2-F	0.010 ± 0.005
**20** 2,4-di Fluoro-PAAH	2-F, 4-F	0.020 ± 0.006
**21** 3-Chloro-4-fluoro-PAAH	3-Cl, 4-F	0.034 ± 0.005
**22** 2-Chloro-PAAH	2-Cl	0.910 ± 0.04
**23** (3-Bromo-PAAH)	3-Br	0.823 ± 0.06

### Pre-steady-state kinetics

**Figure 3.**
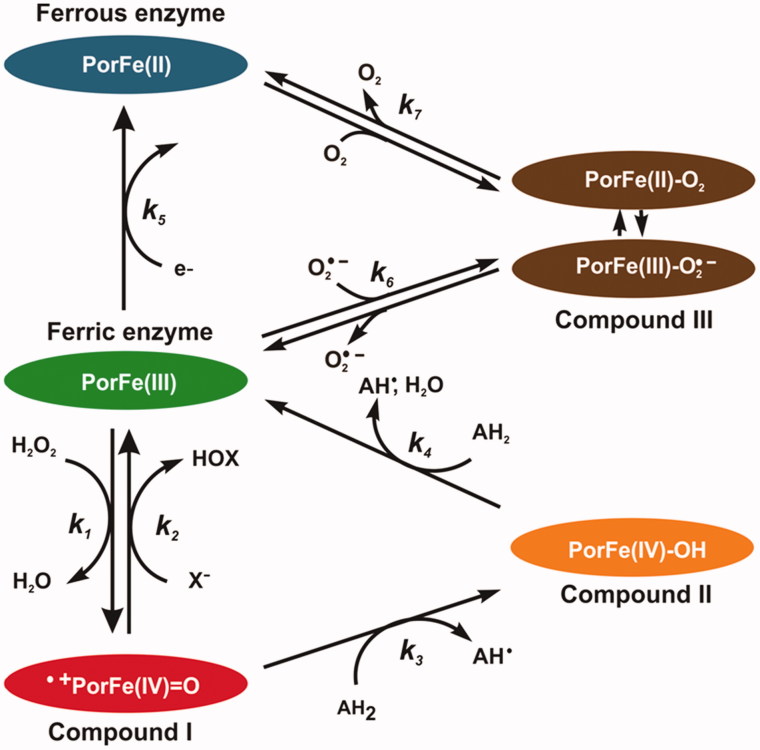
Schematic representation of the halogenation and peroxidase cycles of EPO. Reaction (1): ferric EPO is oxidised by hydrogen peroxide to Compound I (i.e. oxoiron(IV) porphyrin radical cation). Reaction (2): Compound I is directly reduced back to the resting state by halides, thereby releasing hypohalous acid. Reaction (3): Compound I is reduced to Compound II (i.e. protonated oxoiron(IV)) by a one-electron donor. Reaction (4): Compound II is reduced to ferric EPO, thereby oxidising a second substrate molecule. Reaction (5): Ferric enzyme can be reduced to ferrous enzyme. Compound III is formed either from ferric enzyme with superoxide (O_2_^•–^) (Reaction 6), or from ferrous enzyme with O_2_ (Reaction 7) It is a complex of ferrous–dioxygen in resonance with ferric-superoxide, which can dissociate. Reactions 1, 3, and 4 constitute the peroxidase cycle. Reactions (1) and (2) constitute the halogenation cycle.

In order to determine the mechanism of inhibition of EPO with aceto-hydrazide, acetic acid and thioacetic acid derivatives, a pre-steady-state kinetic study was performed with sequential-mixing stopped-flow spectroscopy. Three redox intermediates are relevant in the enzymology of EPO, namely Compound I, Compound II and Compound III (Figure 3). Ferric enzyme is oxidised by hydrogen peroxide into Compound I (*k*_1_), which is directly reduced back to the native state by halides thereby producing hypohalous acid (*k*_2_) those reactions constitute the halogenation cycle. Alternatively, Compound I can also be reduced back by two one-electron reduction steps via Compound II (*k*_3_ and *k*_4_), Figure 3). The ferric enzyme can be reduced to ferrous enzyme (*k*_5_). Compound III can be formed either from ferric enzyme by reversible binding of superoxide (O_2_^–^) (*k*_6_) or from the ferrous enzyme by reversible binding of dioxygen (O_2_) (*k*_7_). A good EPO inhibitor should (i) either efficiently block the entry to the heme cavity or (ii) promote accumulation of Compound II or Compound III, which are outside of the halogenation cycle.

**Figure 4.**
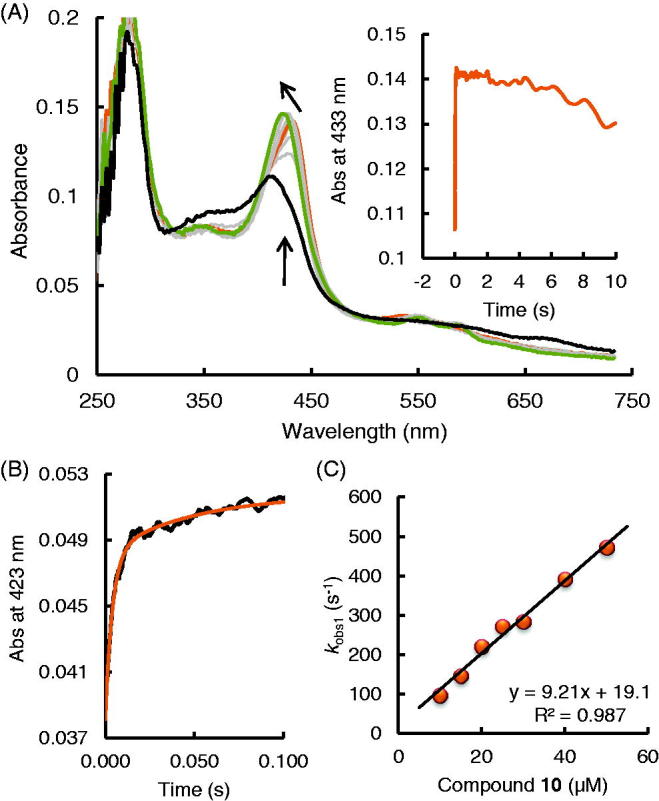
Reaction of EPO Compound I with 2-[(phenyl) amino]-acetohydrazide (compound **10)** (A) Spectral changes upon addition of 10 µM compound **10** to 2 µM Compound I in the sequential-mixing stopped-flow mode. The first spectrum was recorded at 1.3 ms, subsequent spectra at 6.4, 14, 32, 70, 1010, 4666 and 9320 ms. Reaction conditions: 100 mM phosphate buffer, pH 7.0. (B) Typical time trace and double exponential fit of the reaction of Compound I with 10 µM compound **10** followed at 423 nm. Final conditions were 0.5 µM EPO and 0.5 µM H_2_O_2_, 100 mM phosphate buffer, pH 7. (C) Dependency of pseudo-first-order rate constants (*k*_obs(1)_) of Compound I reduction by compound **10**.

Firstly we investigated compound **10**. EPO Compound I can be formed with equimolar concentrations hydrogen peroxide. Compound I decays within the first 10 s to a ferryl/protein radical species^32^. However, in the presence of a compound **10** this reaction is negligible. Figure 4(A) shows the spectral transition when preformed EPO Compound I (2 µM) was mixed with 10 µM compound **10** at pH 7.0 and 25 °C. A very fast direct transition of Compound I (black spectrum) to Compound II (orange spectrum) can be seen, followed by a slow transition to Compound III (green spectrum) with spectral signature (Soret peak at 423 nm and two bands in the visible range at 552 and 588 nm) similar to LPO Compound III^33^. Compound I reduction with compound **10** was measured at 423 nm. At this wavelength both the Compound II formation and the transition to Compound III could be followed.

The time traces could be fitted with a double-exponential equation, were the pseudo-first-order rate constant *k*_obs(1)_ was dependent on the concentration of compound **10.** This represents the reduction of Compound I to Compound II, whereas the following slow transition (*k*_obs(2)_) was independent of the concentration of compound **10** and represents the conversion of Compound II to Compound III. Potting *k*_obs(1)_ against the compound **10** concentration the apparent second-order rate constant (9.2 × 10^6^ M^−1^s^−1^) for Compound I reduction could be obtained. The rate constant for transition Compound II - Compound III was in the order of 1.2 s^−1^. From the reaction scheme (Figure 3) it is clear that Compound III can only be formed from ferric enzyme (*k*_6_) or from ferrous enzyme (*k*_7_) with activated oxygen or dioxygen respectively. It seems that the PAAH radicals which are generated during Compound I and II reduction react faster with ferric enzyme and reduces it to ferrous EPO than Compound II reduction occurs. Or PAAH radical activates dioxygen with can react with ferric enzyme to generate again Compound III. This is similar to the mechanism-based 4-aminobenzoic acid hydrazide (ABAH) inhibitor of myeloperoxidase that is oxidised to radical intermediates that cause enzyme inactivation. ABAH readily reduced Compound I and reacted also with Compound II, but it is a poor peroxidase substrate because the free radicals formed during peroxidation converted the enzyme to Compound III. It was proposed that ABAH destroys the heme prosthetic groups of the enzyme by reducing a ferrous enzyme and Compound III-complex^34^.

**Figure 5.**
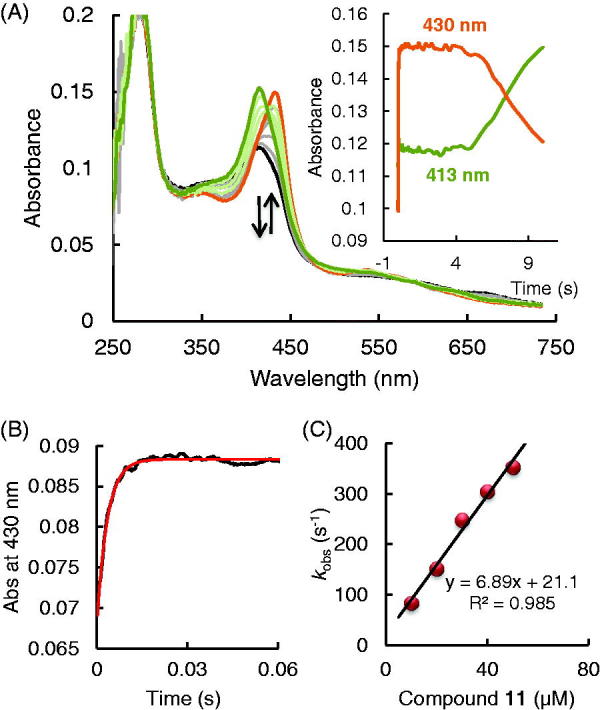
Reaction of EPO Compound I with 2-[(4-chlorophenyl) amino]-acetic acid (compound **11**) (A) Spectral changes upon addition of 10 µM compound **11** to 2 µM Compound I in the sequential-mixing stopped-flow mode. The first spectrum was recorded at 1.3 ms, subsequent spectra were recorded at time points indicated in the spectrum. Reaction conditions: 100 mM phosphate buffer, pH 7.0, Inset shows the time traces at 413 and 430 nm. (B) Typical time trace and single exponential fit of the reaction of Compound I with 30 µM compound **11** followed at 430 nm. Final conditions were 0.5 µM EPO and 0.5 µM H_2_O_2_, 100 mM phosphate buffer, pH 7. (C) Dependency of pseudo-first-order rate constants (*k*_obs_) of Compound I reduction by compound **11**.

Figure 5 demonstrates the kinetics of the reactions between compound **11** and EPO Compound I and Compound II. The inhibitor acted as one electron donors for both redox intermediates of EPO. Figure 5 depicts the direct transition from Compound I to Compound II followed by the Compound II reduction to ferric enzyme-mediated by oxidation of compound **11**. From the first part of the biphasic reaction *k*_obs_ value showed a linear dependence on inhibitor concentration and apparent second-order rate constant (*k*_3_) of 6.9 × 10^6^ M^−1^s^−1^ was calculated (Figure 5(C)). The high intercept clearly demonstrated that Compound II was not stable but further transformed in a direct monophasic transition from Compound II to ferric enzyme-mediated by compound **11** with a clear isosbestic point at 424 nm. This is clear by examining the constant absorbance at 430 nm (inset to Figure 5) after the initial phase. The length of this phase is strictly correlated with the amount of H_2_O_2_ present in the system. After complete hydrogen peroxide consumption, Compound II was converted to the ferric enzyme (Figure 5) in a concentration-dependent manner. The apparent second-order rate constant (*k*_4_) was 1.1 × 10^4^ M^−1^ s^−1^, which suggest that Compound II reduction was the rate-limiting step in the peroxidase cycle. Compound **11** was an excellent electron donor for Compound I, but even poorer substrates for Compound II therefore, keeping the enzyme outside of the halogenation cycle and decreasing the release of hypobromous acid. In contrast to compound **10**, which seems to be an irreversible inhibitor, compound **11** can be considered as reversible inhibitor.

**Figure 6.**
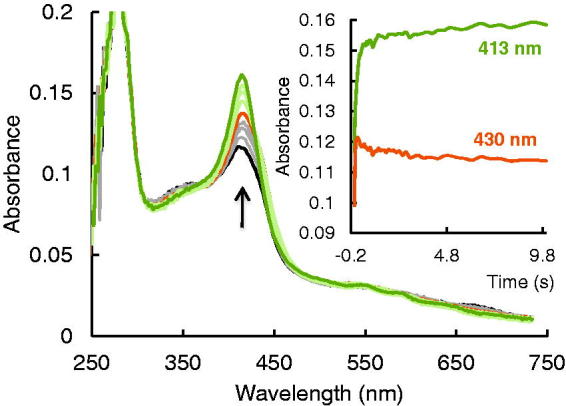
Reaction of EPO Compound I with 2-[(4-chlorophenyl)thio]-acetohydrazide (compound **12**) (A) Spectral changes upon addition of 10 µM compound **12** to 2 µM Compound I in the sequential-mixing stopped-flow mode. The first spectrum was recorded at 1.3 ms, subsequent spectra at 0.0064, 0.012, 0.027, 0.073, 0.157, 0.303, 1010, and 9988 ms. Reaction conditions: 100 mM phosphate buffer, pH 7.0, Inset shows the time traces at 413 and 430 nm.

A similar situation occurs with 2-[(4-chlorophenyl) thio]-aceto-hydrazide (compound **12**). The inhibitor acted as one electron donor to both redox intermediates Compound I and Compound II of EPO. Figure 6 shows the direct transition from Compound I to Compound II followed by a fast Compound II reduction to ferric enzyme-mediated by oxidation compound **12**. The apparent second-order rate constants are 4.3 × 10^6^ M^−1^s^−1^ for Compound I formation and 1.4 × 10^5^ M^−1^s^−1^ for Compound II reduction. Compound II reduction of compound **12** is much higher in contrast to compound **11.** Therefore Compound II cannot accumulate and the originating ferric EPO can enter again both the halogenation and peroxidase cycle. But at higher compound **12** concertation the peroxidase cycle dominates the halogenation cycle and inhibits the hypobromous acid formation.

## Conclusion

Within this study, a pharmacophore modeling and virtual screening approach led to the discovery of several potent inhibitors of eosinophil peroxidase (EPO) derived from the 2-(phenyl)amino-aceto-hydrazides scaffold. The ligand-based pharmacophore models created were experimentally validated and therefore constitute valuable tools for the selection of compounds for biological testing. The identified EPO inhibitors might possibly contribute to the development of novel lead compounds as starting points for the development of new medicines for the treatment of inflammatory and neurodegenerative diseases caused by eosinophilic disorders such as bronchial asthma, inflammatory bowel diseases or ALS. Future studies will address the analysis of the pharmacological profile of the most potent compounds, such as compound **10** (2-[(4-chlorophenyl) amino]-aceto-hydrazide) and compound **19** (2-[(2-fluorophenyl) amino]-aceto-hydrazide), with IC_50_-values of 21 nM and 10 nM, in more detail. Additionally, our pharmacophore models will serve in future *in silico* screening experiments of further compound databases to search for novel EPO inhibitors from other sources, e.g. natural products, and will also be used to guide our medicinal chemistry lead optimisation program in this field.

## Supplementary Material

Supplemental Material
