# Supplementary material for: Pharmacophore-based discovery of 2-(phenylamino)aceto-hydrazides as potent eosinophil peroxidase (EPO) inhibitors
Source: J Enzyme Inhib Med Chem. 2018 Oct 4;33(1):1529–36. doi: 10.1080/14756366.2018.1512598 (PMC6179059; doi:10.1080/14756366.2018.1512598)
Supplement: Supplemental Material [file IENZ_A_1512598_SM8200.zip › IENZ_1512598_Supplementary Materials/Supplemental Information A_marked.pdf]

Supplemental Information A

Pharmacophore-based Discovery of 2-(Phenylamino)Aceto-Hydrazides as Potent Eosinophil Peroxidase (EPO) Inhibitors

Daniela Schuster[a], Martina Zederbauer[b], Thierry Langer[c], Andreas Kubin[b*] Paul G. Furtmüller[d*]

[a] *Paracelsus Medical Private University Salzburg, Institute of Pharmacy, Department of Pharmaceutical and Medicinal Chemistry, Strubergasse 13, 5020 Salzburg, Austria*

[b] *Planta Naturstoffe, Erlgasse 48, A-1120 Vienna, Austria*

[c] *Division of Drug Design and Medicinal Chemistry, Department of Pharmaceutical Chemistry, Faculty of Life Sciences, University of Vienna, Althanstr. 14, 1090 Vienna, Austria*

[d] *University of Natural Resources and Life Sciences, Department of Chemistry, Division of Biochemistry, Muthgasse 18, 1190 Vienna, Austria*

* corresponding authors: Paul G. Furtmüller, University of Natural Resources and Life Sciences, Department of Chemistry, Division of Biochemistry, Muthgasse 18, 1190 Vienna, Austria, email: paul.furtmueller@boku.ac.at;

Andreas Kubin, Planta Naturstoffe, Erlgasse 48, A-1120 Vienna, Austria, email: a.kubin@planta.at

Supplementary Figure S1: IC 50 plots of inhibition of EPO halogenation activity. Figure A-N shows the effect of the different inhibitors on the halide oxidation of 100 mM bromide by 20 nM EPO and 200 µM hydrogen peroxide. Reactions were carried out in 100 mM phosphate buffer, pH 7, at 25 °C, Data are means of triplicate experiments. The inhibitory effect of each inhibitor was measured by determining the concentration at which it inhibited oxidation of MCD by 50% (IC50).

A

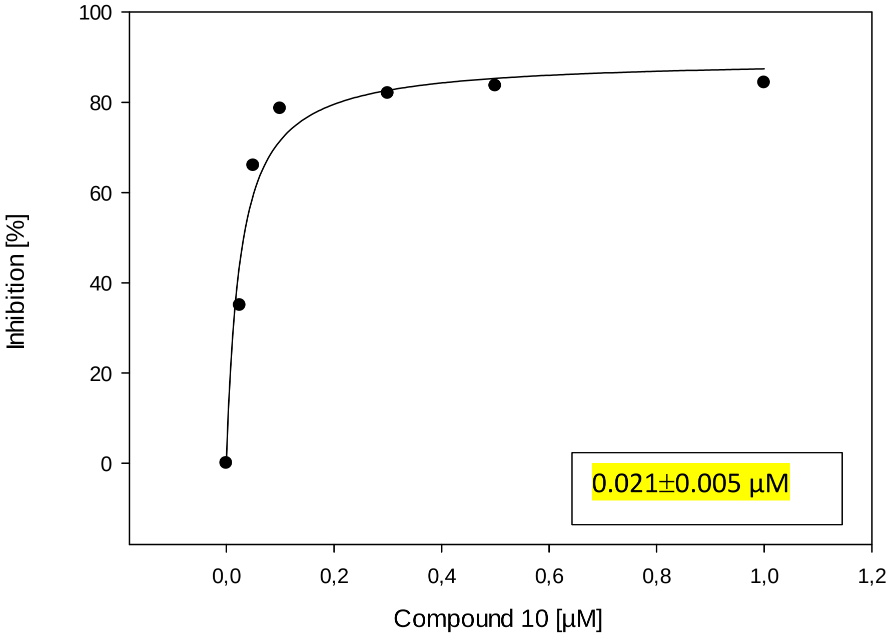

B

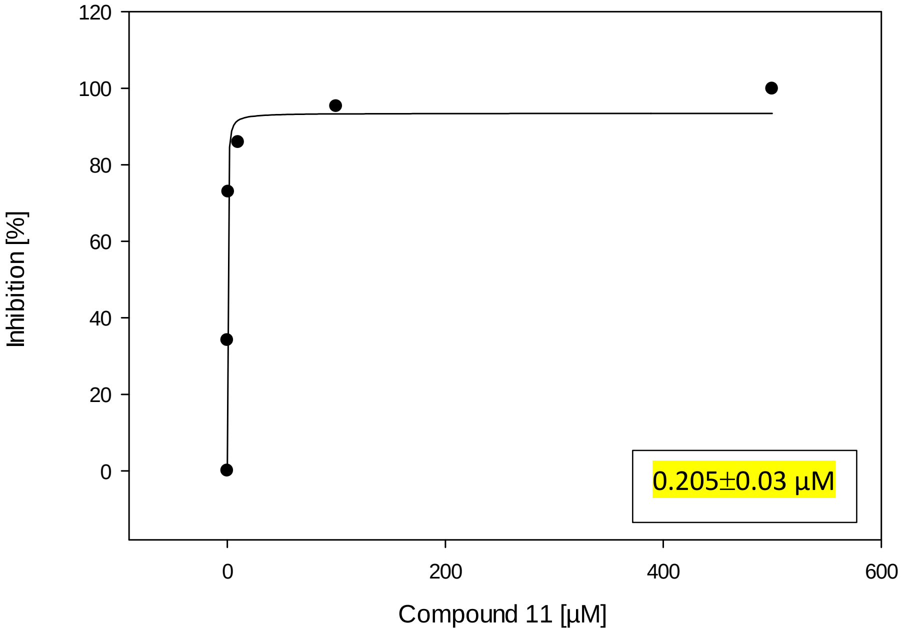
120
100
80
60
40
20
0
Inhibition [%]
0
200
400
600
Compound 11 [µM]
0.205±0.03 µM

C

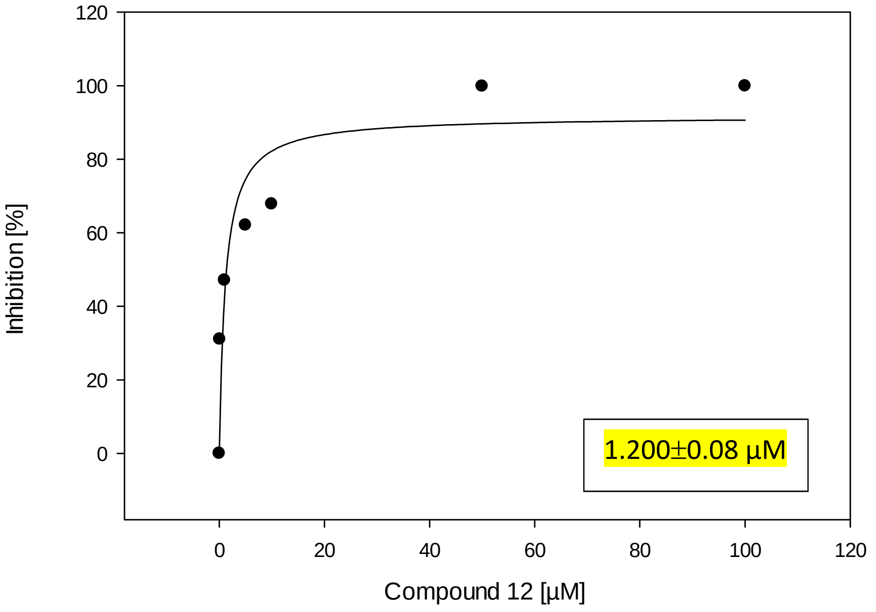
120
100
80
60
40
20
0
Inhibition [%]
0
20
40
60
80
100
120
Compound 12 [µM]
1.200±0.08 µM

D

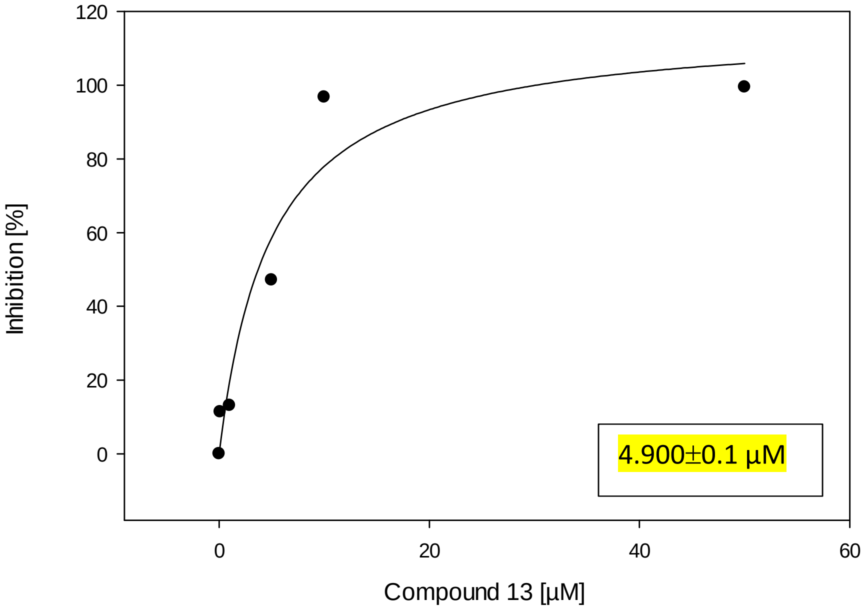

120
100
80
60
40
20
0
Inhibition [%]
0
20
40
60
Compound 13 [µM]
4.900±0.1 µM

E

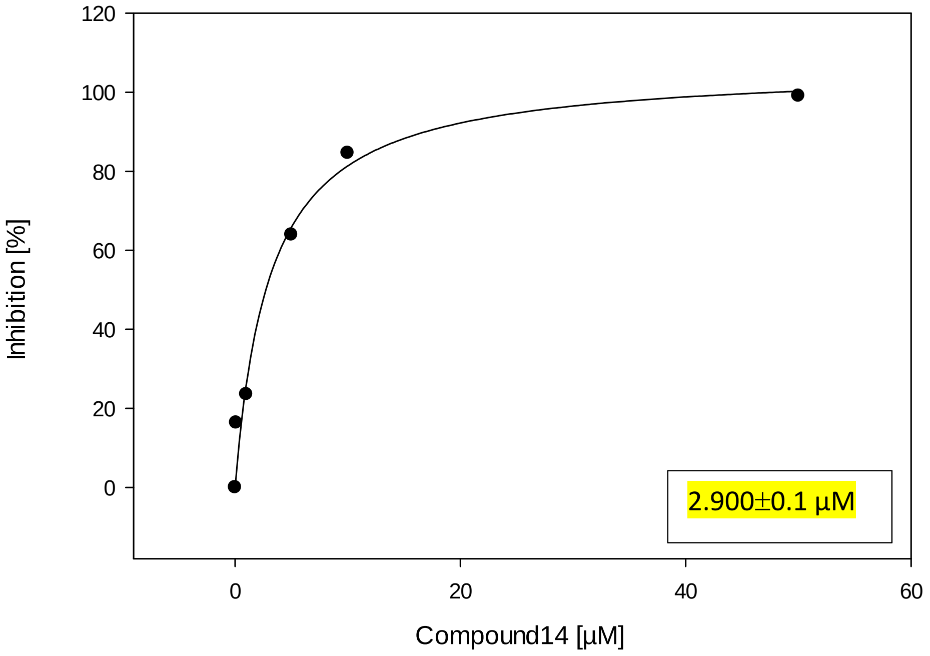

120
100
80
60
40
20
0
Inhibition [%]
0
20
40
60
Compound14 [µM]
2.900±0.1 µM

F

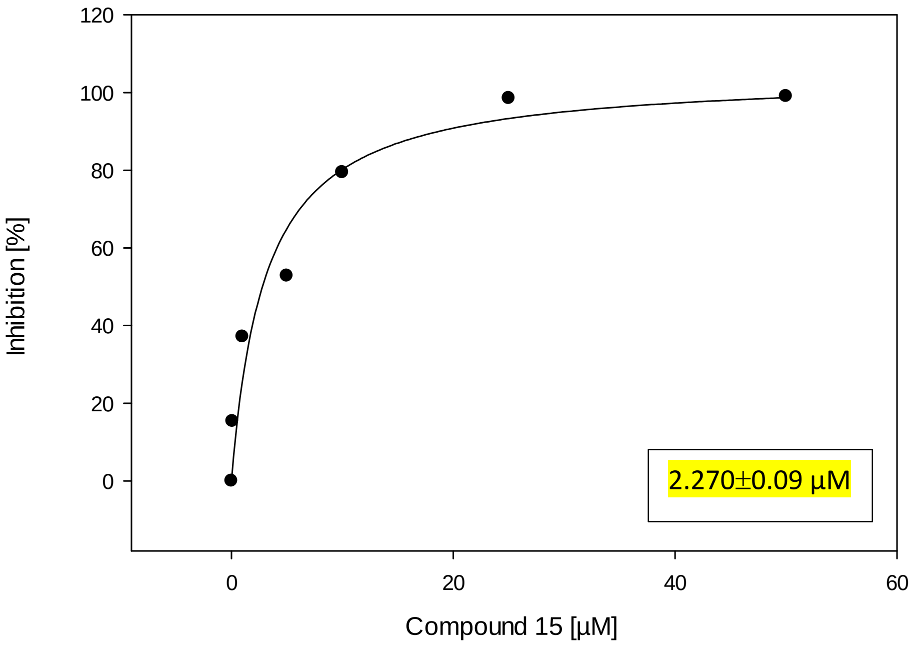

Inhibition [%]
120
100
80
60
40
20
0
0
20
40
60
Compound 15 [µM]
2.270±0.09 µM

G

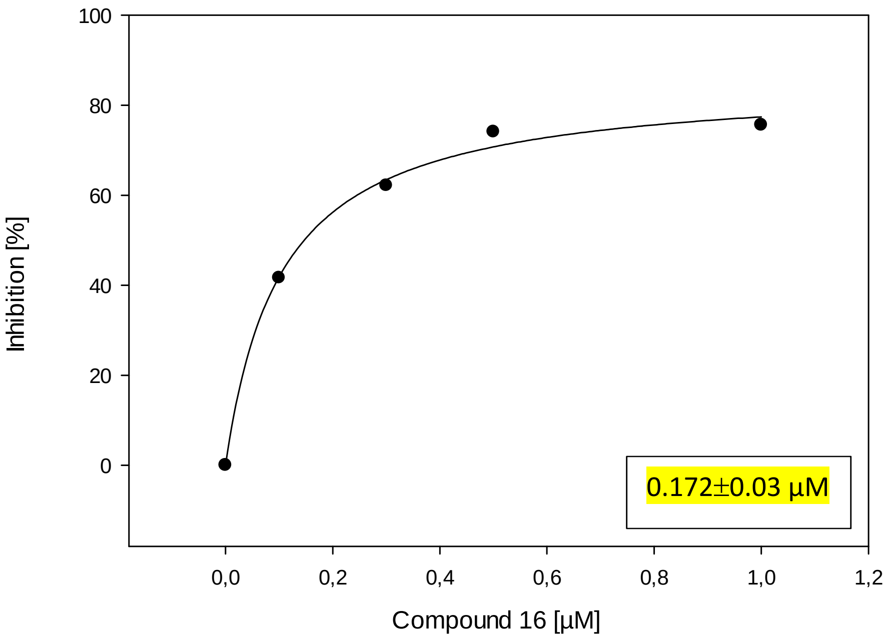

Inhibition [%]
100
80
60
40
20
0
0,0
0,2
0,4
0,6
0,8
1,0
1,2
Compound 16 [µM]
0.172±0.03 µM

H

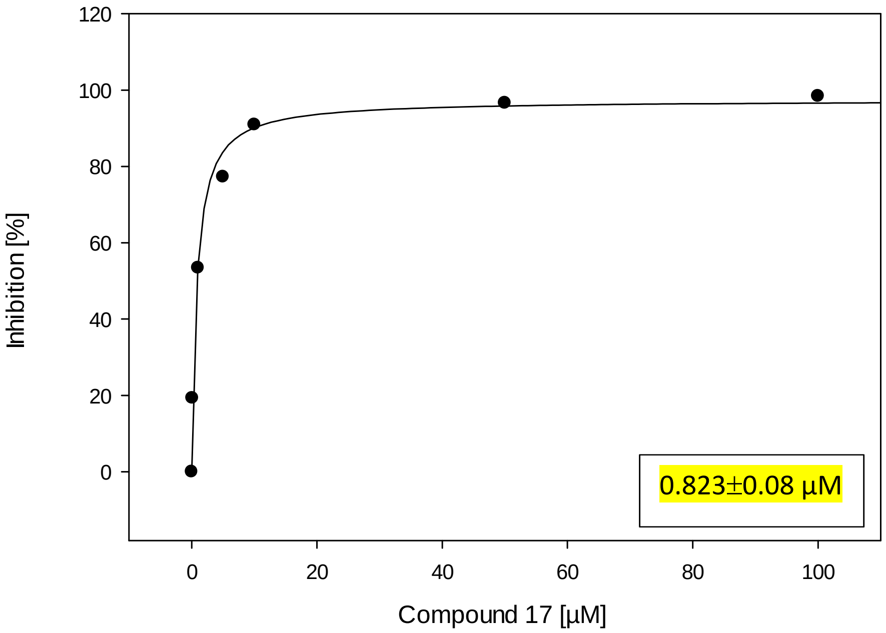

120
100
80
60
40
20
0
Inhibition [%]
0.823±0.08 µM
0
20
40
60
80
100
Compound 17 [µM]

I

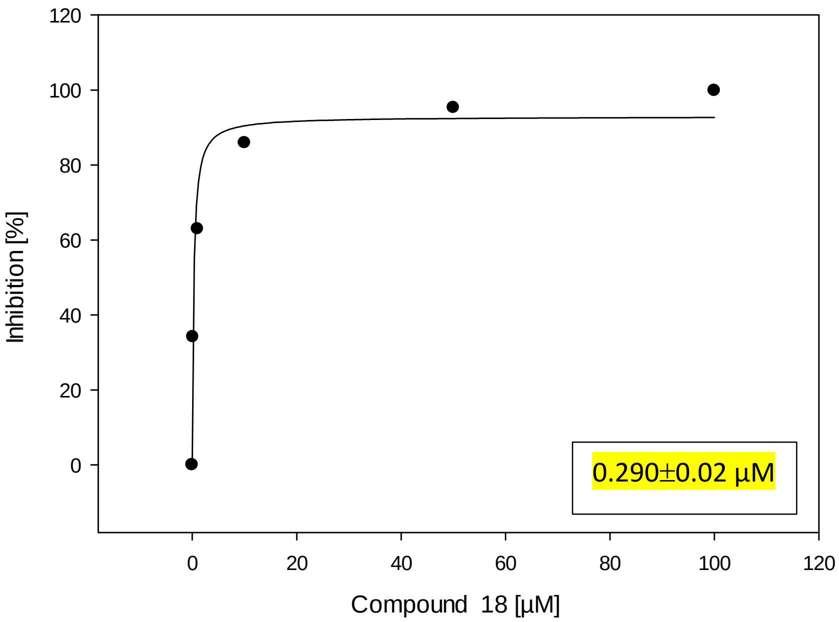

120
100
80
60
40
20
0
Inhibition [%]
0.290±0.02 µM
0
20
40
60
80
100
120
Compound 18 [µM]

J

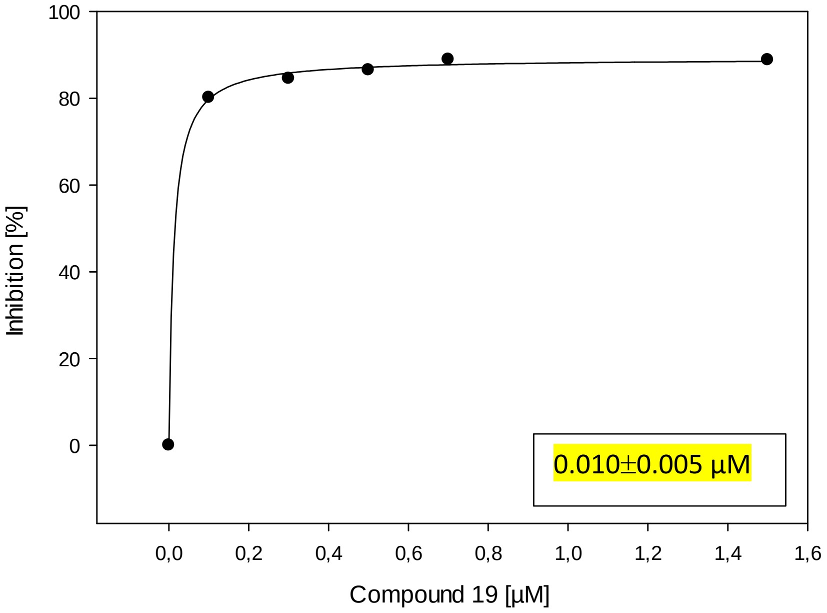
100
80
60
40
20
0
Inhibition [%]
0.010±0.005 µM
0,0
0,2
0,4
0,6
0,8
1,0
1,2
1,4
1,6
Compound 19 [µM]

K

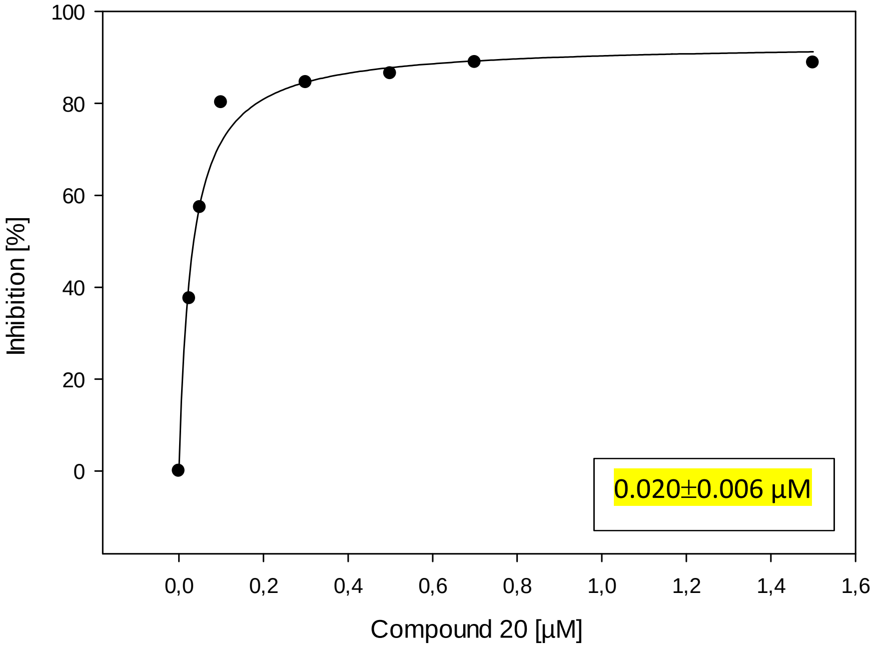
100
80
60
40
20
0
Inhibition [%]
0.020±0.006 µM
0,0
0,2
0,4
0,6
0,8
1,0
1,2
1,4
1,6
Compound 20 [µM]

L

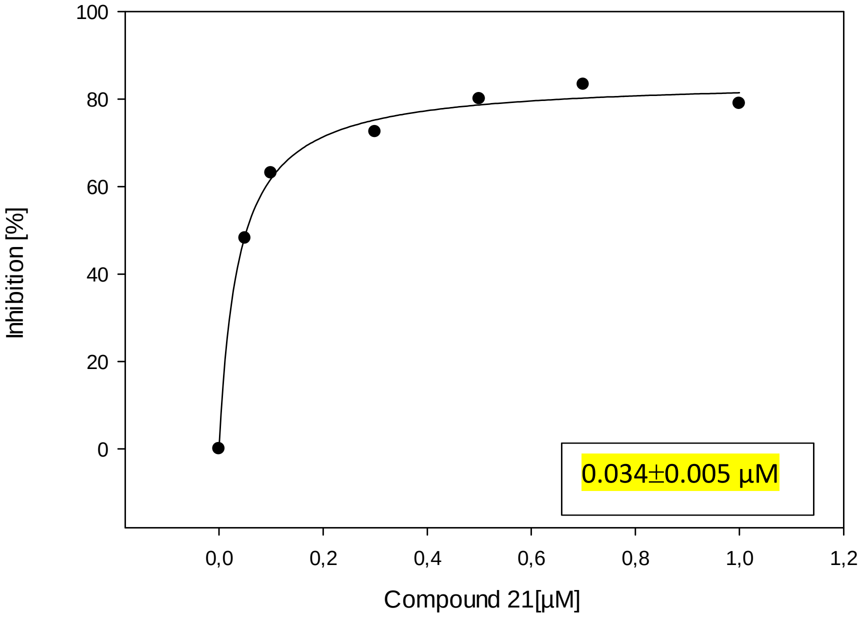

100
80
60
40
20
0
Inhibition [%]
0.034±0.005 µM
0,0
0,2
0,4
0,6
0,8
1,0
1,2
Compound 21[µM]

M

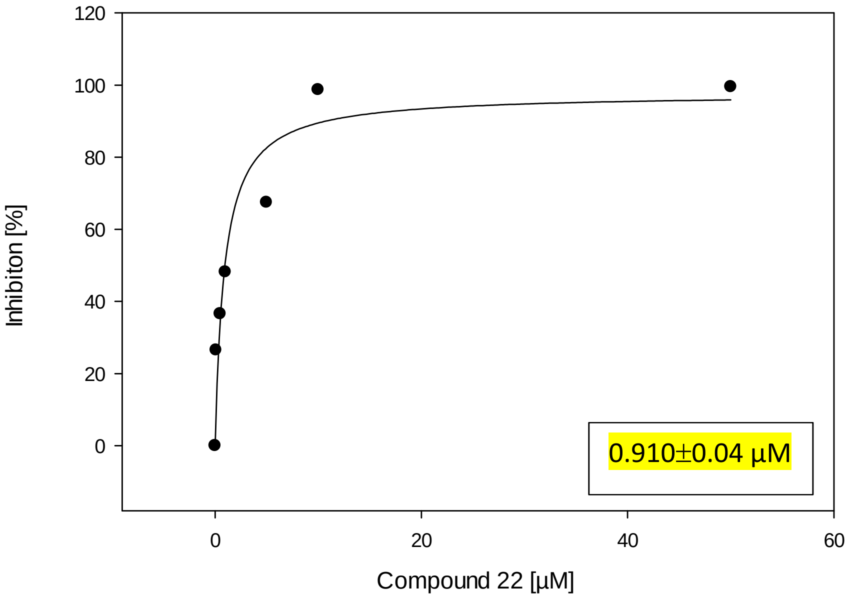

120
100
80
60
40
20
0
Inhibiton [%]
0.910±0.04 µM
0
20
40
60
Compound 22 [µM]

N

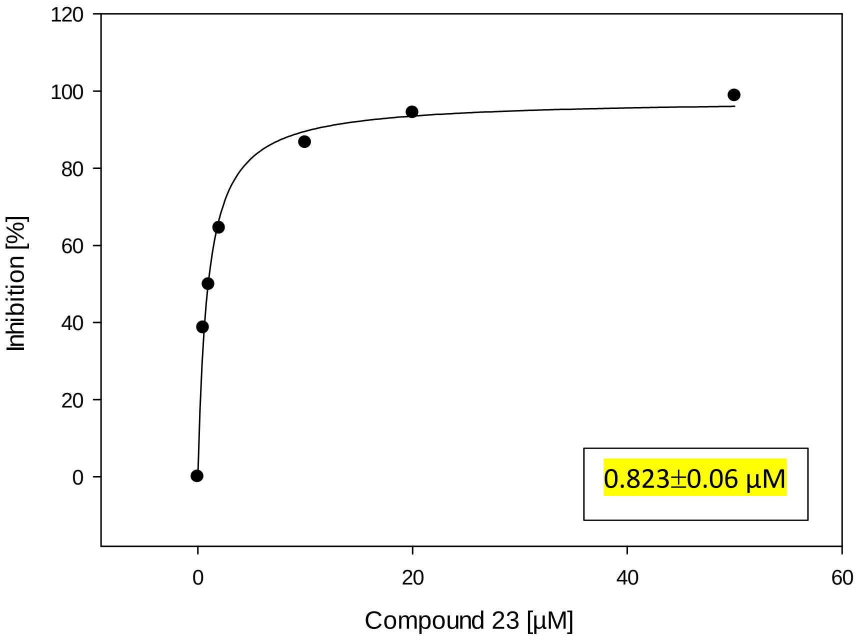
Inhibition [%]
120
100
80
60
40
20
0
0
20
40
60
Compound 23 [µM]
0.823±0.06 µM
