# Supplementary material for: Pharmacophore-based discovery of 2-(phenylamino)aceto-hydrazides as potent eosinophil peroxidase (EPO) inhibitors
Source: J Enzyme Inhib Med Chem. 2018 Oct 4;33(1):1529–36. doi: 10.1080/14756366.2018.1512598 (PMC6179059; doi:10.1080/14756366.2018.1512598)

Supplemental Information B

Pharmacophore-based Discovery of 2-(Phenylamino)Aceto-Hydrazides as Potent Eosinophil Peroxidase (EPO) Inhibitors

Daniela Schuster[a], Martina Zederbauer[b], Thierry Langer[c], Andreas Kubin[b*] Paul G. Furtmüller[d*]

[a] *Paracelsus Medical Private University Salzburg, Institute of Pharmacy, Department of Pharmaceutical and Medicinal Chemistry, Strubergasse 13, 5020 Salzburg, Austria*

[b] *Planta Naturstoffe, Erlgasse 48, A-1120 Vienna, Austria*

[c] *Division of Drug Design and Medicinal Chemistry, Department of Pharmaceutical Chemistry, Faculty of Life Sciences, University of Vienna, Althanstr. 14, 1090 Vienna, Austria*

[d] *University of Natural Resources and Life Sciences, Department of Chemistry, Division of Biochemistry, Muthgasse 18, 1190 Vienna, Austria*

* corresponding authors: Paul G. Furtmüller, University of Natural Resources and Life Sciences, Department of Chemistry, Division of Biochemistry, Muthgasse 18, 1190 Vienna, Austria, email: paul.furtmueller@boku.ac.at;

Andreas Kubin, Planta Naturstoffe, Erlgasse 48, A-1120 Vienna, Austria, email: a.kubin@planta.at

NMR and MS Analysis of the Lead Substances

MS analysis: MS spectra were redorded on a Bruker maXis HD™, ESI-qTOF

AW/EPO/002, 3.86 mg, 2-[(4-Chlorophenyl)amino]ethanhydrazid,
MS Analysis: MW = 199,60 g/mol

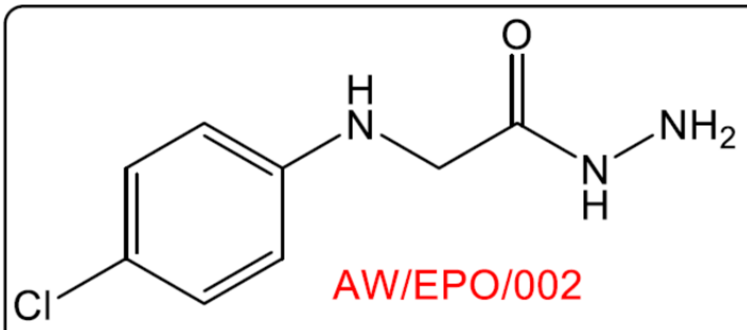

AW/EPO/002

2-[(4-Chlorophenyl)amino]ethanhydrazid
2-[(4-chlorophenyl)amino]acetohydrazide
Chemical Formula: $C_8H_{10}ClN_3O$
Exact Mass: 199,05

AW/EPO/PD01, 2,86 mg, N-[(2-Fluorophenyl)amino]acetic acid,
MS Analysis: MW = 191,2 g/mol

AW/EPO/PD01

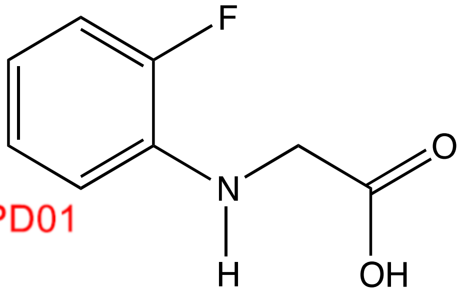

N-[(2-Fluorophenyl)amino]acetic acid
(2-fluorophenyl)glycine (PIN)
Chemical Formula: $C_8H_8FNO_2$
Exact Mass: 169,05

AW/EPO/ PD02,4,62 mg, N-[(Chlorophenyl)amino]acetic acid,
MS Analysis: MW = 207,6 g/mol

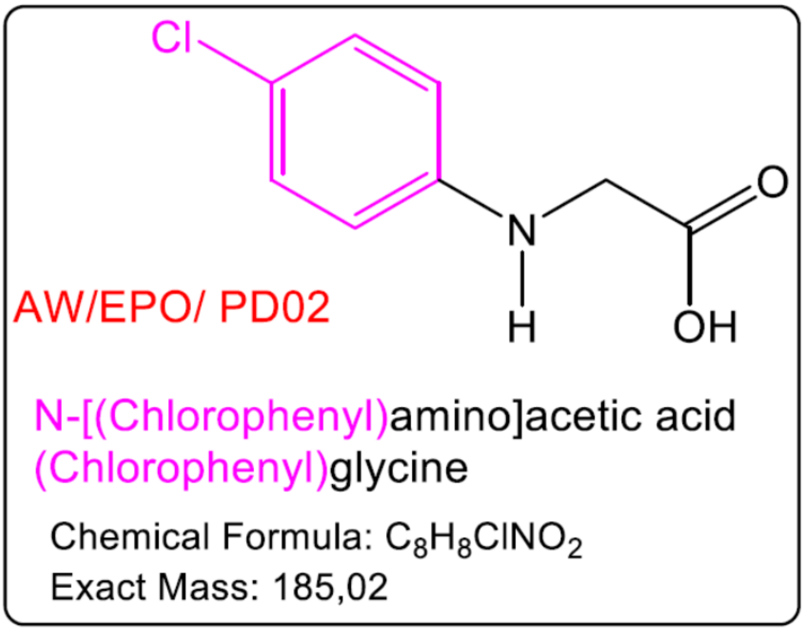

AW/EPO/ PD06, 3.76 mg, 2-[(4-Chlorophenyl)sulfanyl]acetohydrazid,
MS Analysis: MW = 216,69 g/mol

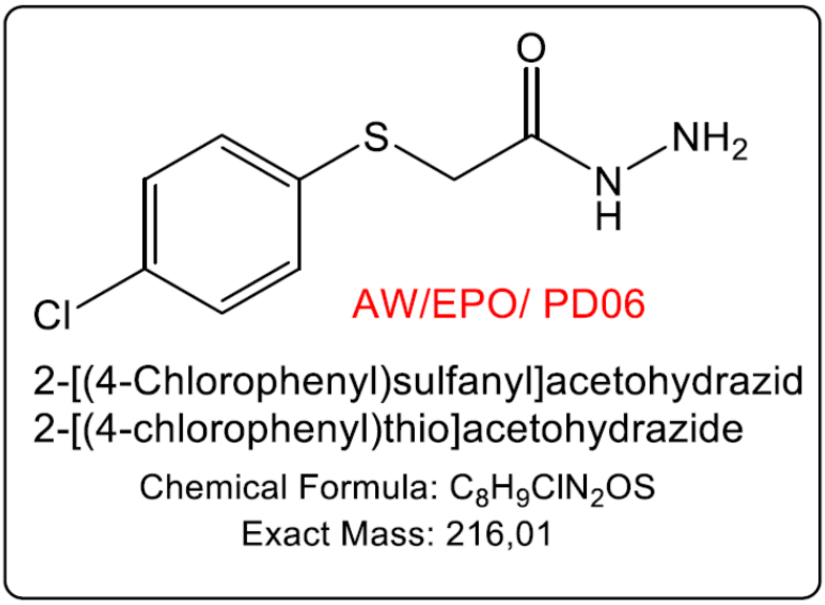

AW/EPO/003, 2,70 mg, 2-[(2-Fluorophenyl)amino]ethanhydrazid,
MS Analysis: MW = 183,19 g/mol

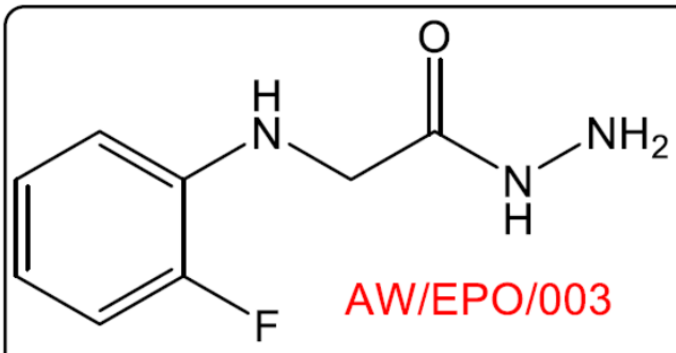

AW/EPO/003

2-[(2-Fluorophenyl)amino]ethanhydrazid

Chemical Formula: $C_8H_{10}FN_3O$

Exact Mass: 183,08

NMR analysis. NMR spectra were recorded on a Bruker Avance 500 NMR spectrometer (UltraShield) using a 5 mm switchable probe
(TCI Prodigy Kryo-probe head, 5 mm, tripelresonance-invers-detection probe head) with z axis gradients and automatic tuning and matching accessory (Bruker BioSpin). The resonance frequency for ^{1}H NMR was 500.13 MHz and for ^{13}C NMR 125.75 MHz. All measurements were performed for a solution in fully deuterated chloroform or methanol at 298 K. Standard 1D and gradient-enhanced (ge) 2D experiments, like double quantum filtered (DQF) COSY, NOESY, HSQC, and HMBC, were used as supplied by the manufacturer. Chemical shifts are referenced internally to the residual, non-deuterated solvent signal for chloroform ^{1}H (δ 7.26 ppm) and to the carbon signal of the solvent for chloroform ^{13}C (δ 77.00 ppm).

AWEPO002 in cdcl3 (APT) 2.11.2016

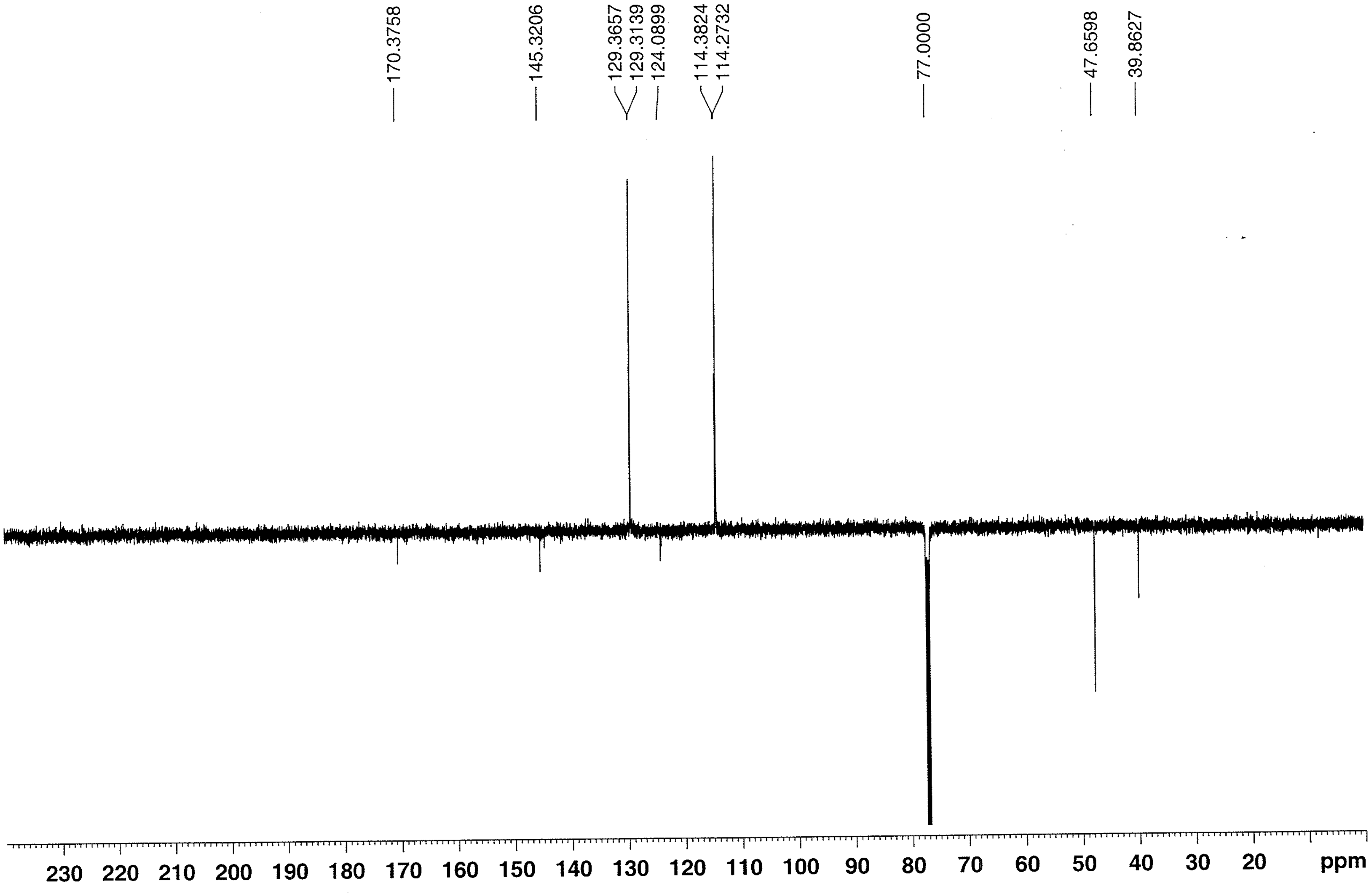

AWEP0002 in cdcl3 (APT) 2.11.2016

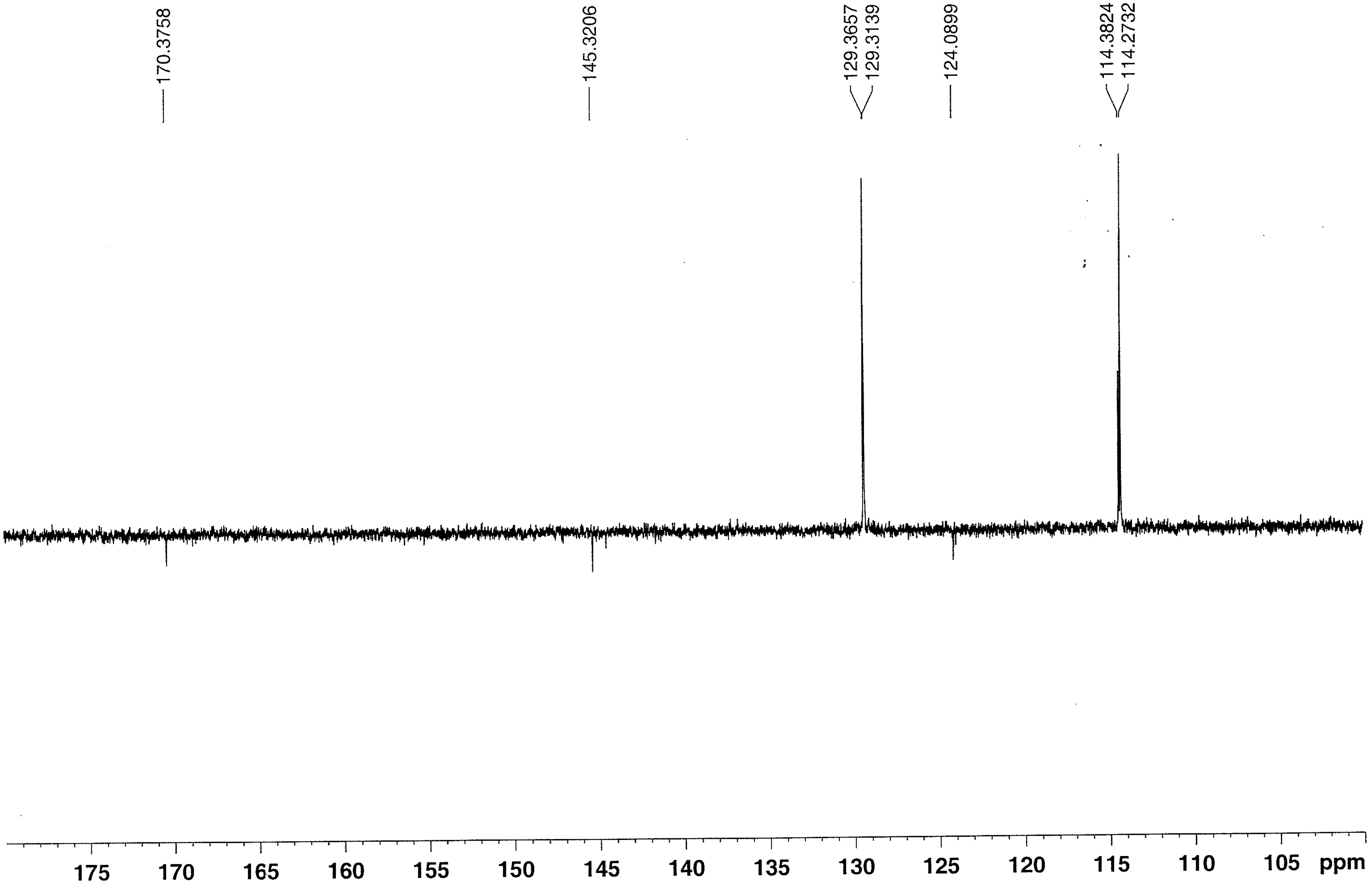

AWEPO002 in cdcl3 (APT) 2.11.2016

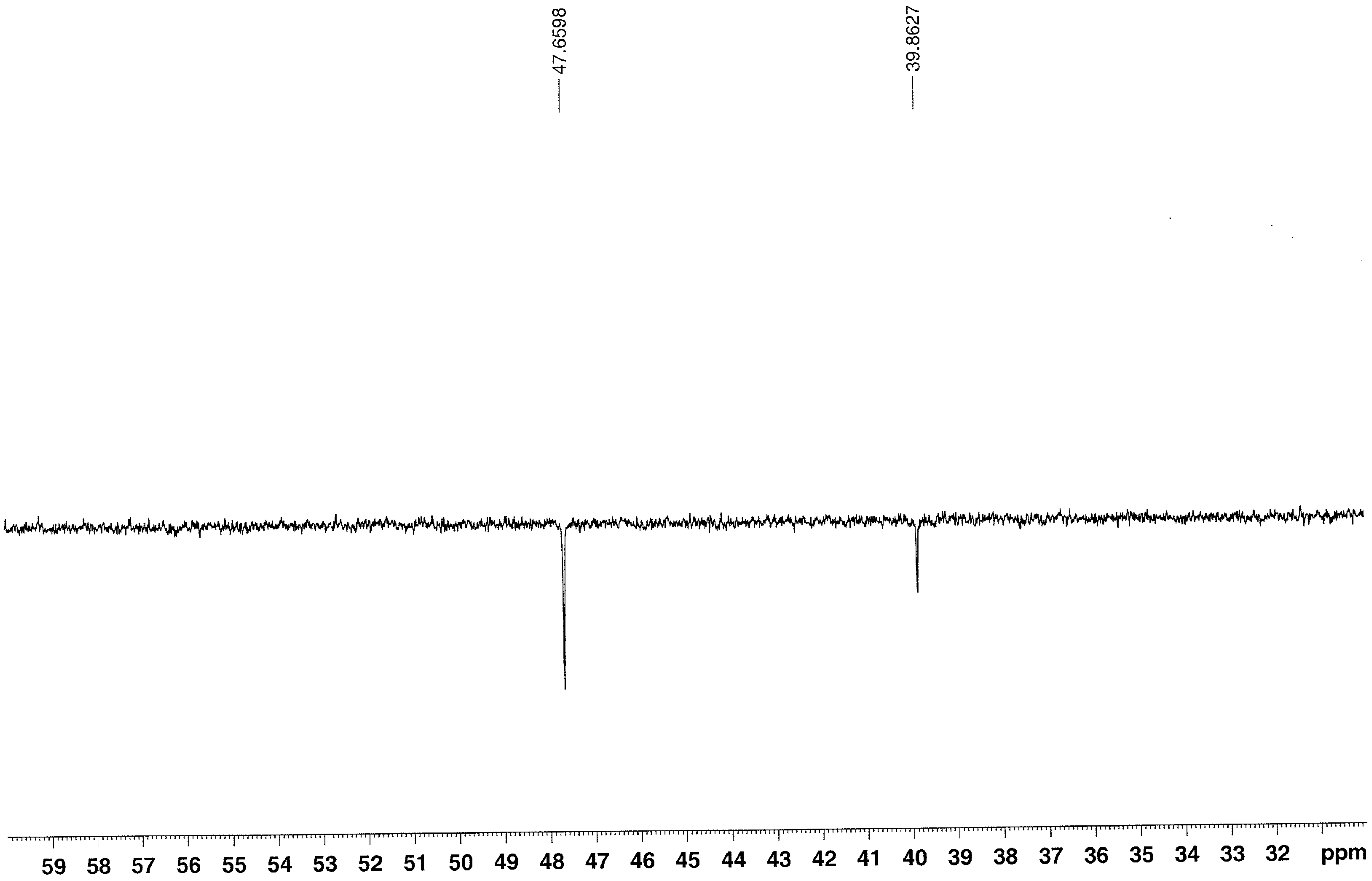

AWEPO003 in cdcl3 (APT) 2.11.2016

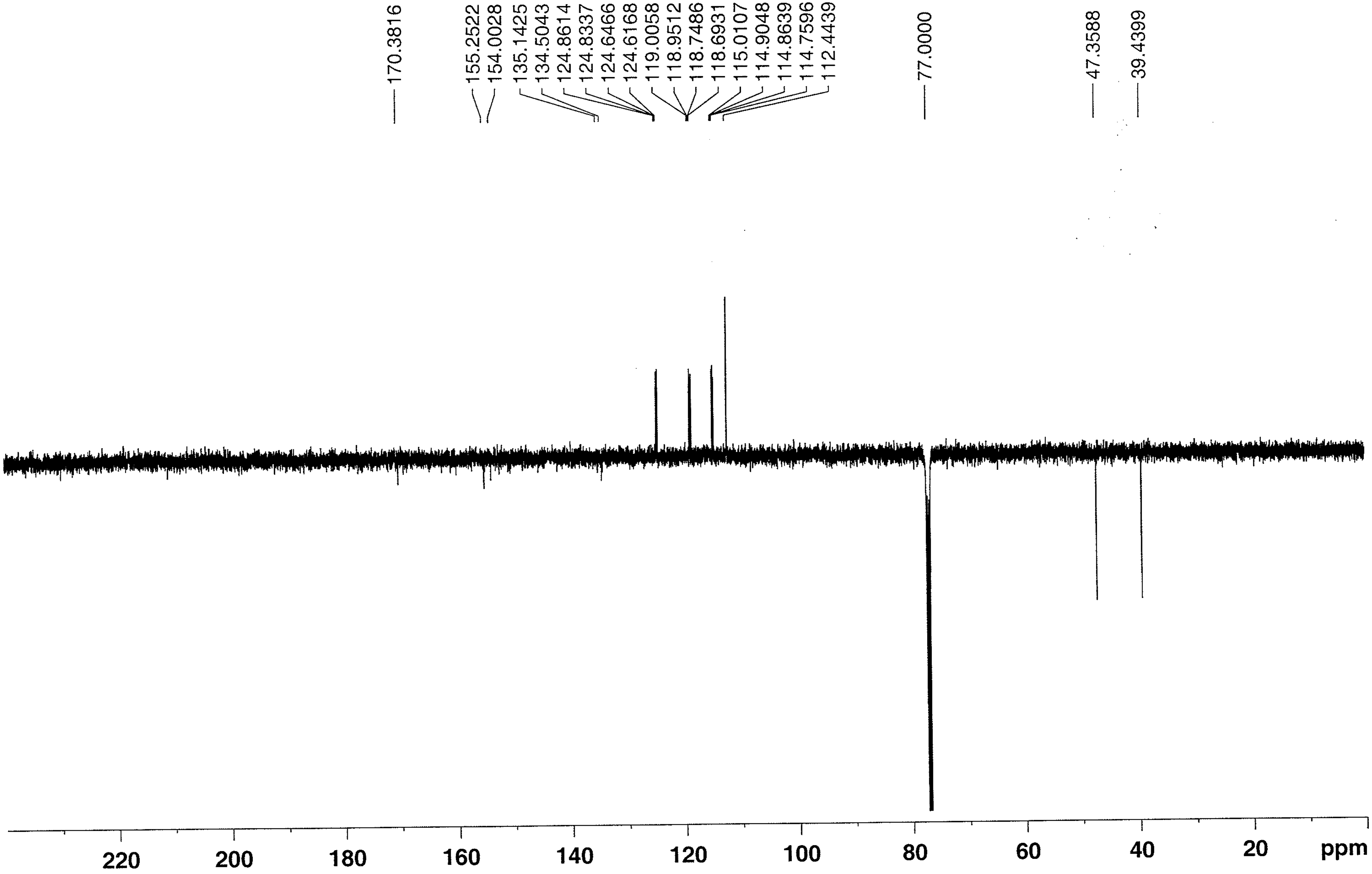

AWEPO003 in cdcl3 (APT) 2.11.2016

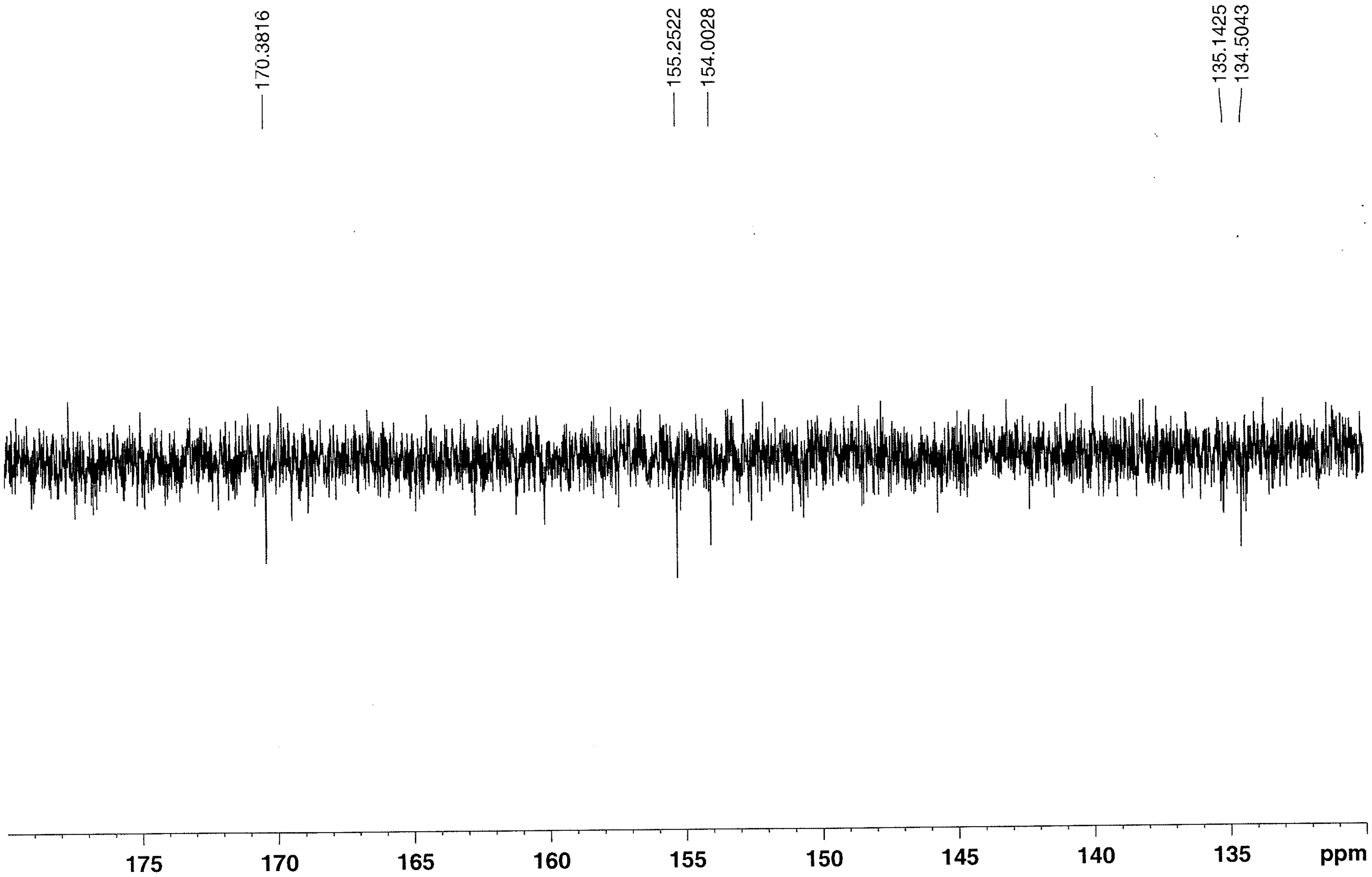

AWEPO003 in cdcl3 (APT) 2.11.2016

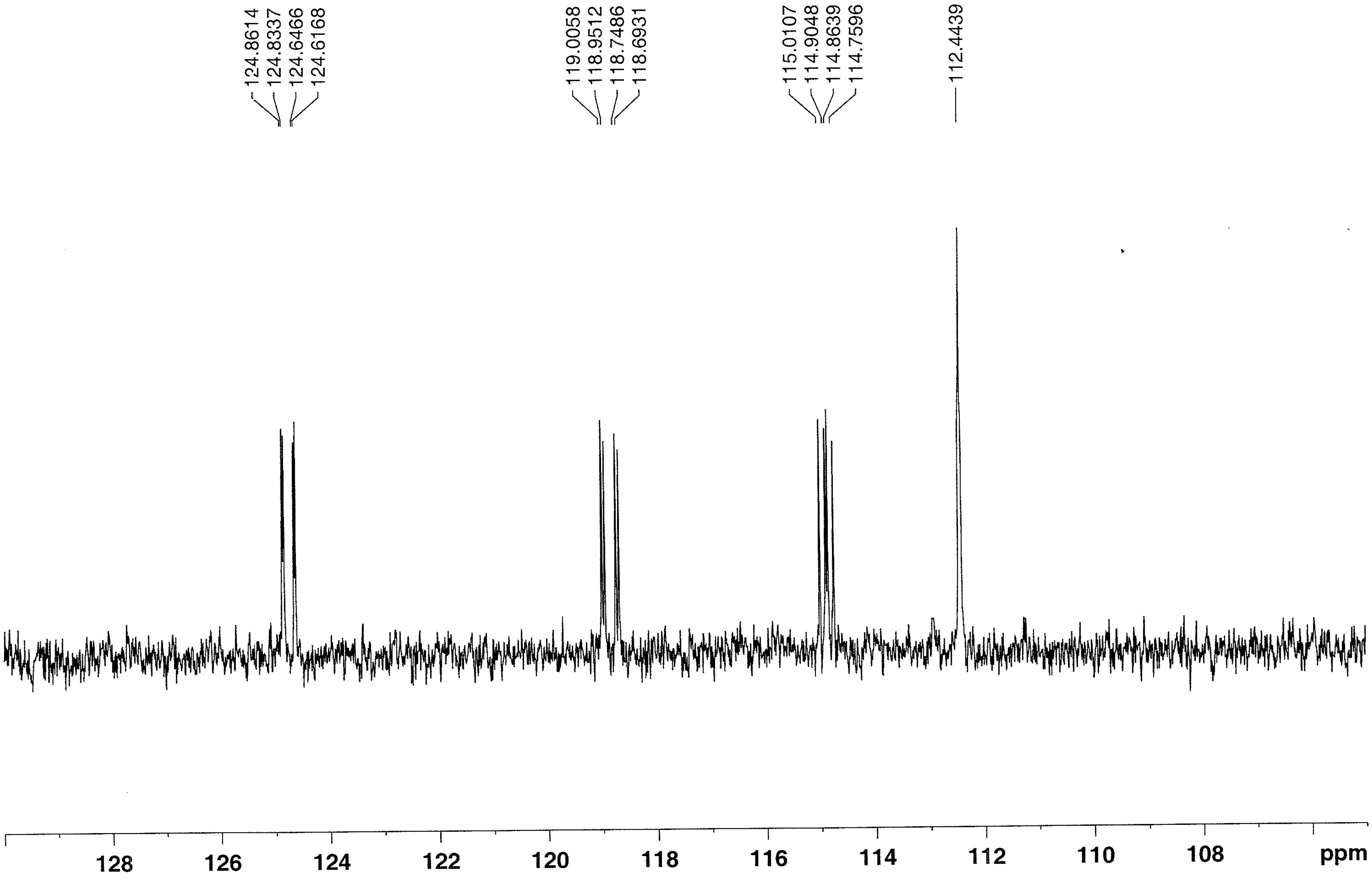

AWEPO003 in cdcl3 (APT) 2.11.2016

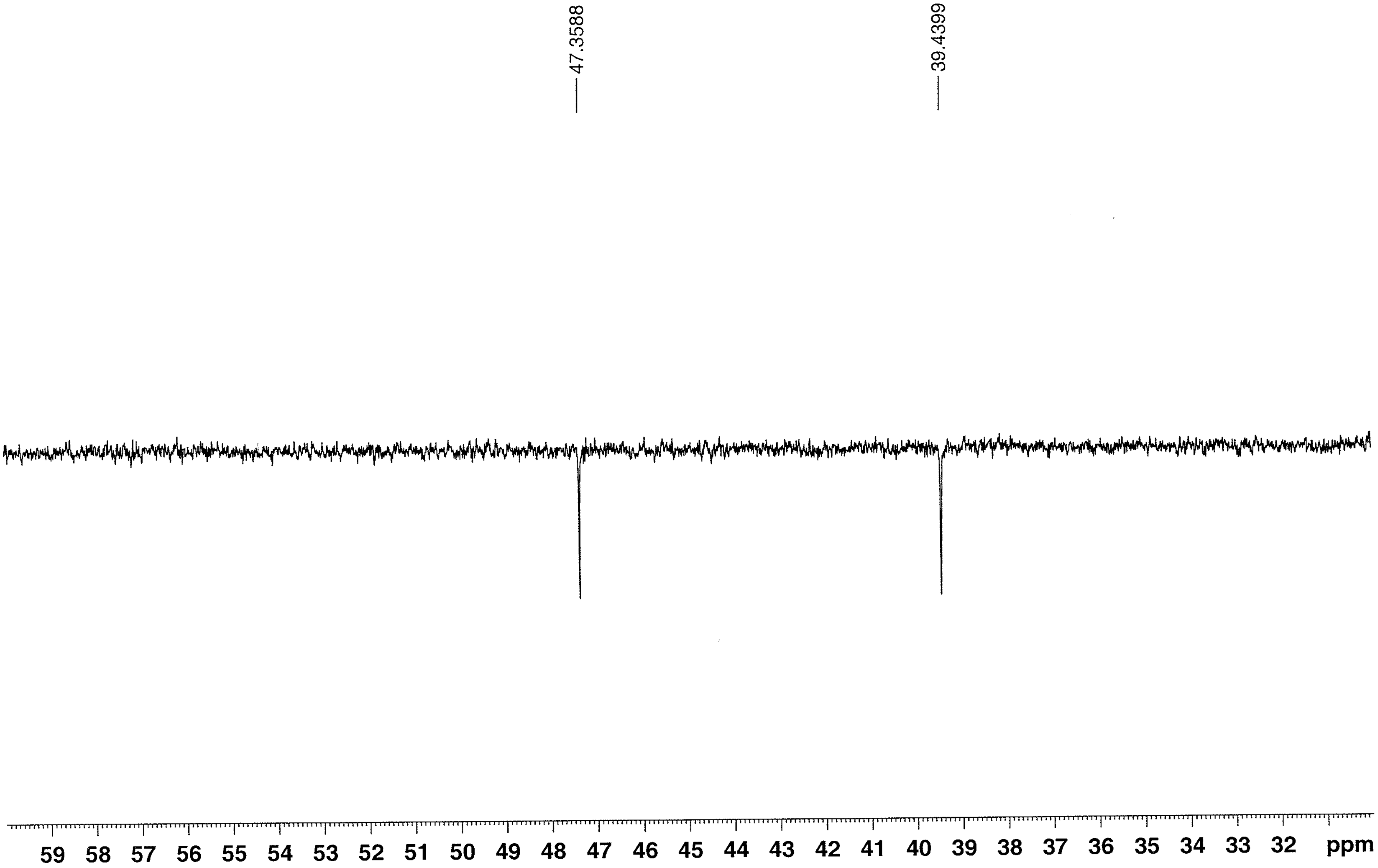

AWEPOpd01 in cdcl3 (APT) 2.11.2016

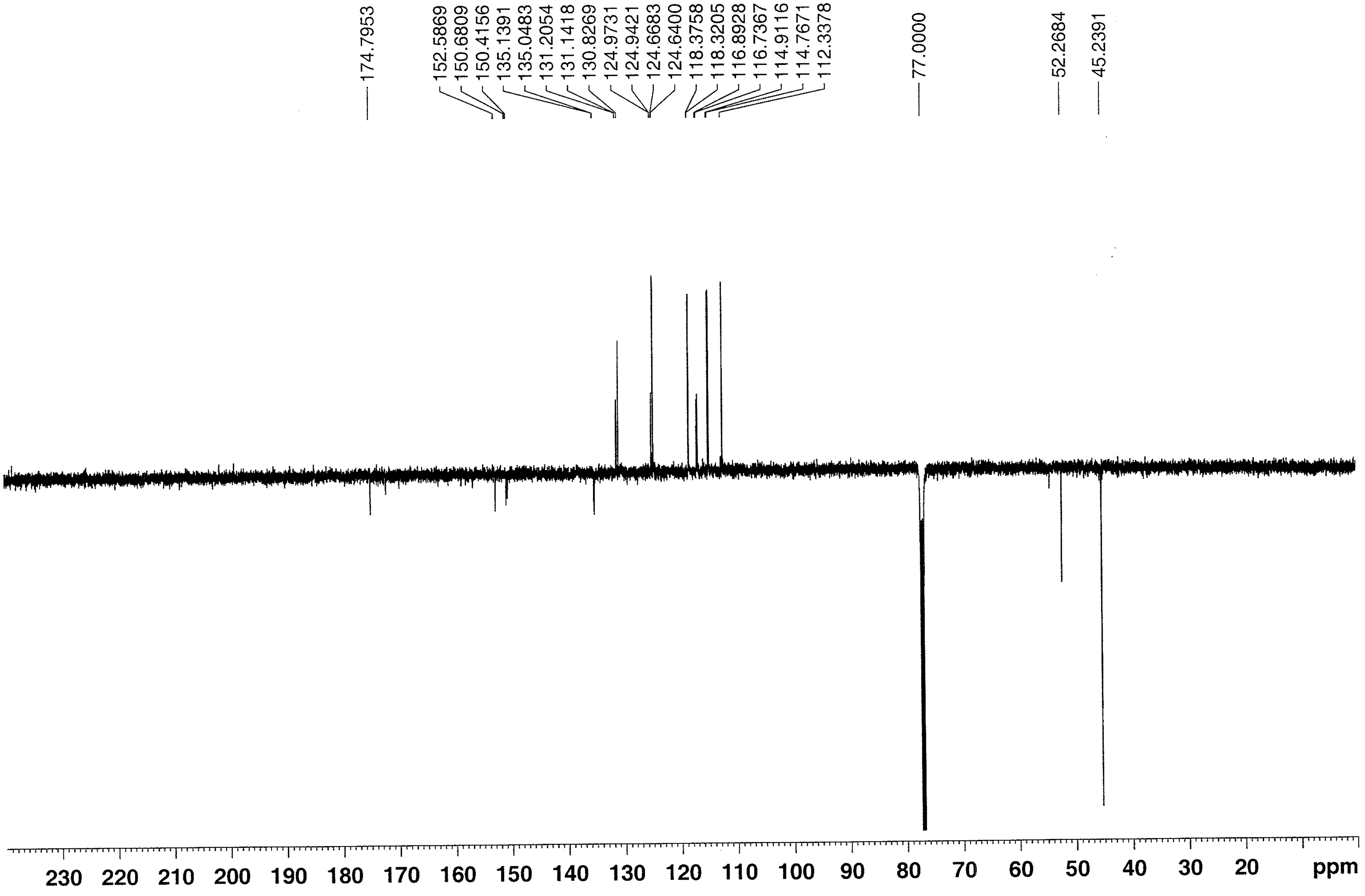

AWEPOpd01 in cdcl3 (APT) 2.11.2016

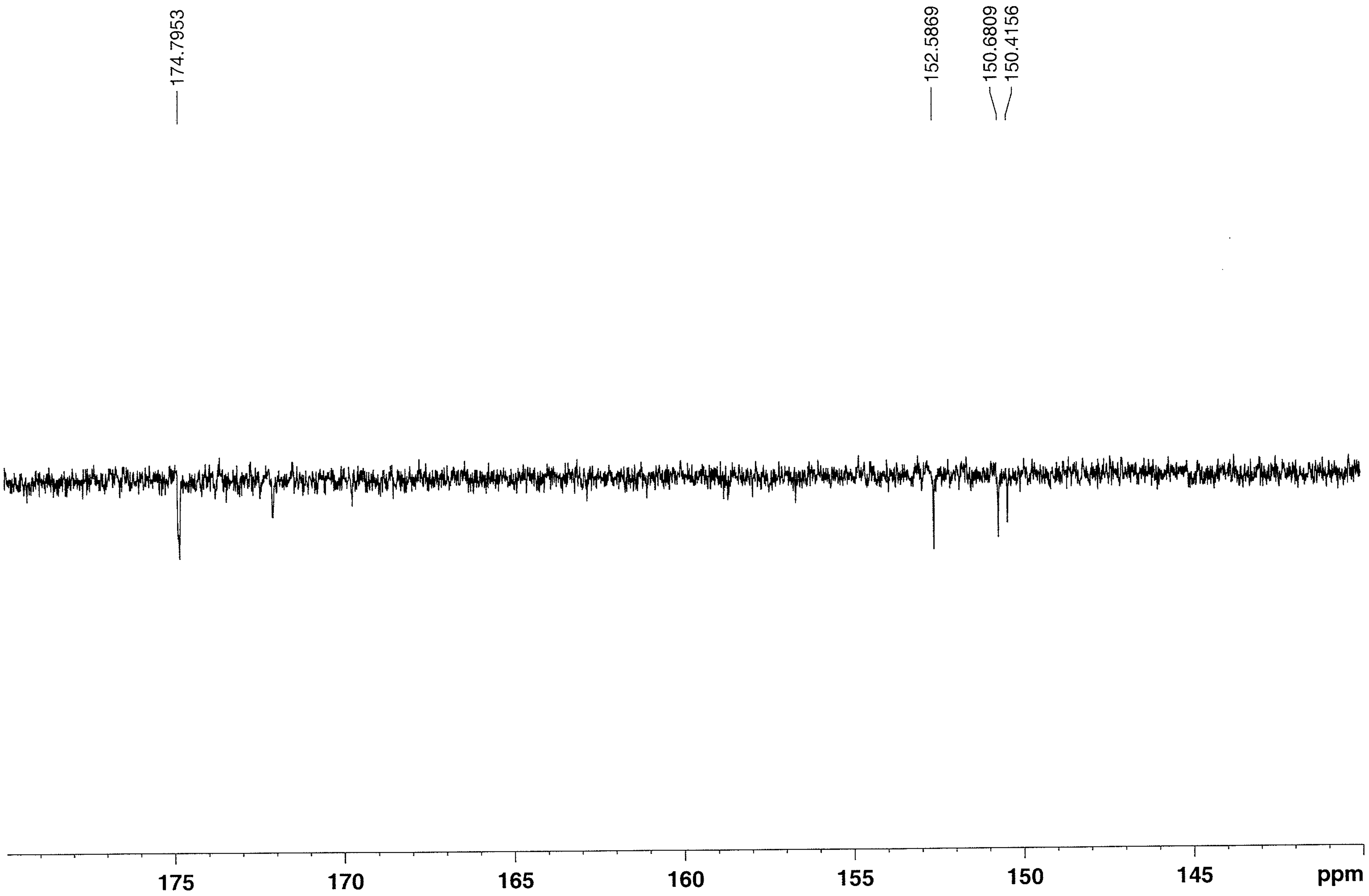

AWEPOpd01 in cdcl3 (APT) 2.11.2016

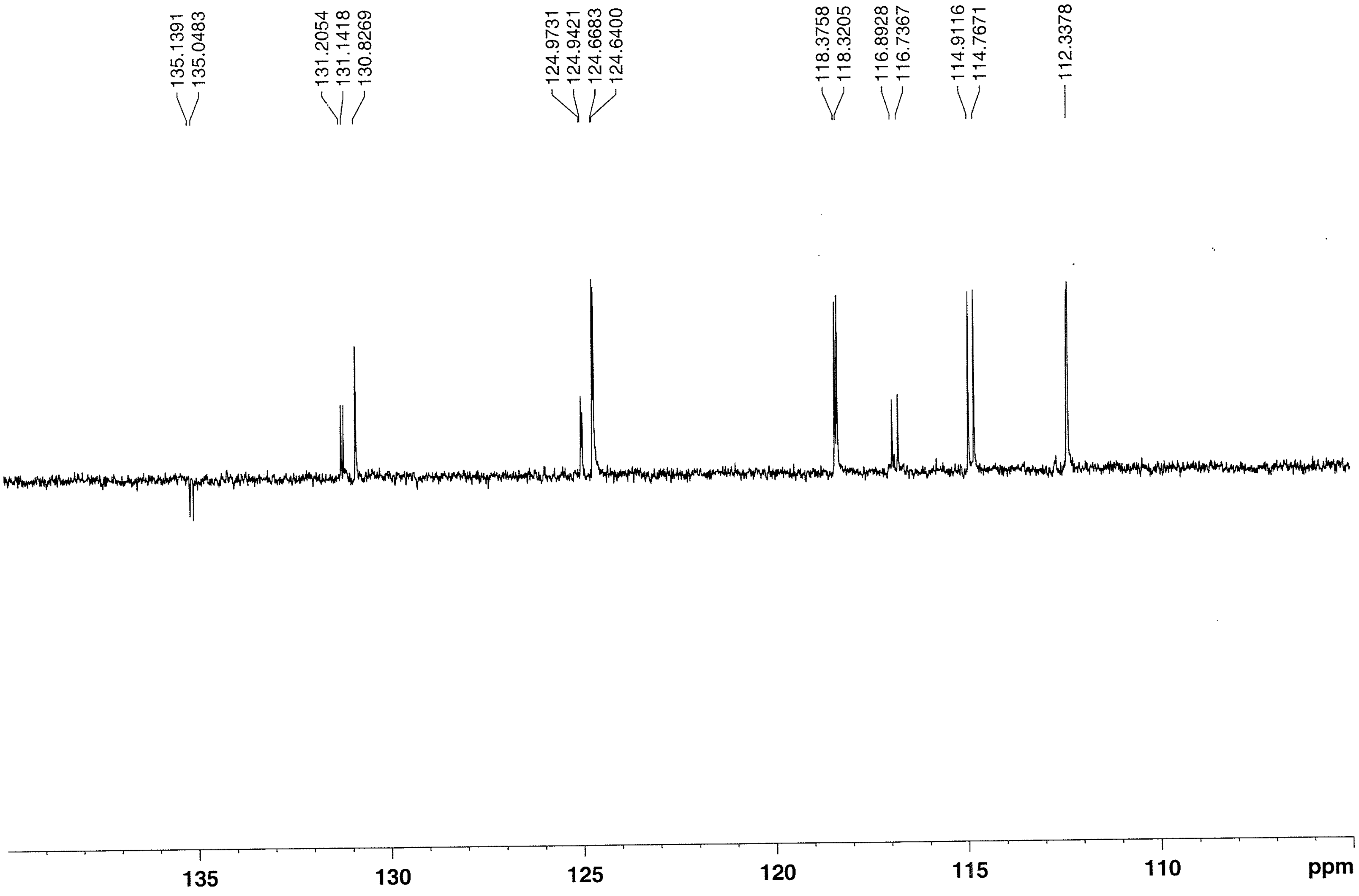

AWEPOpd01 in cdcl3 (APT) 2.11.2016

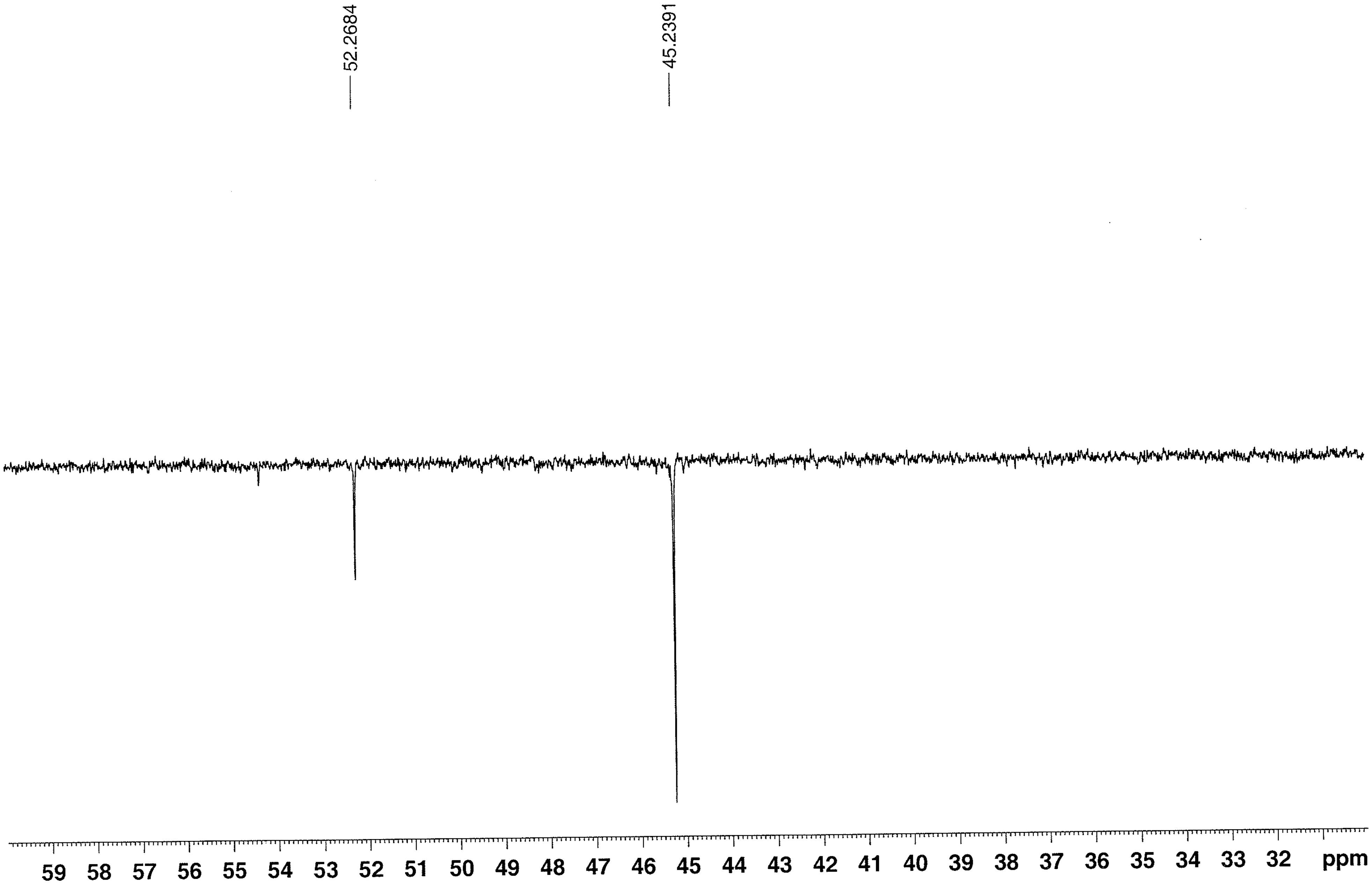

AWEPOpd02 in cdcl3 (APT) 2.11.2016

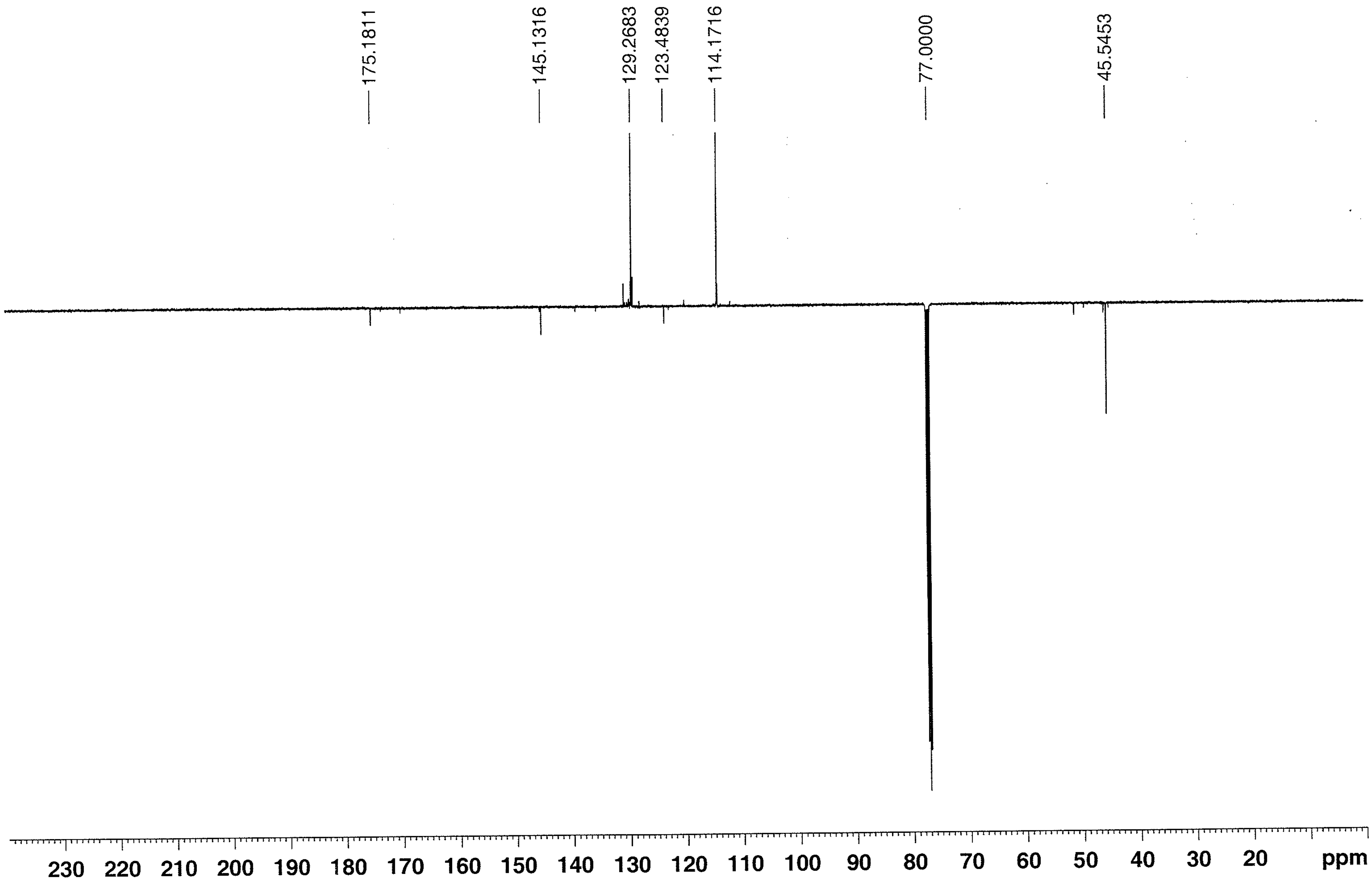

AWEPOpd06 in cdcl3 (APT) 2.11.2016

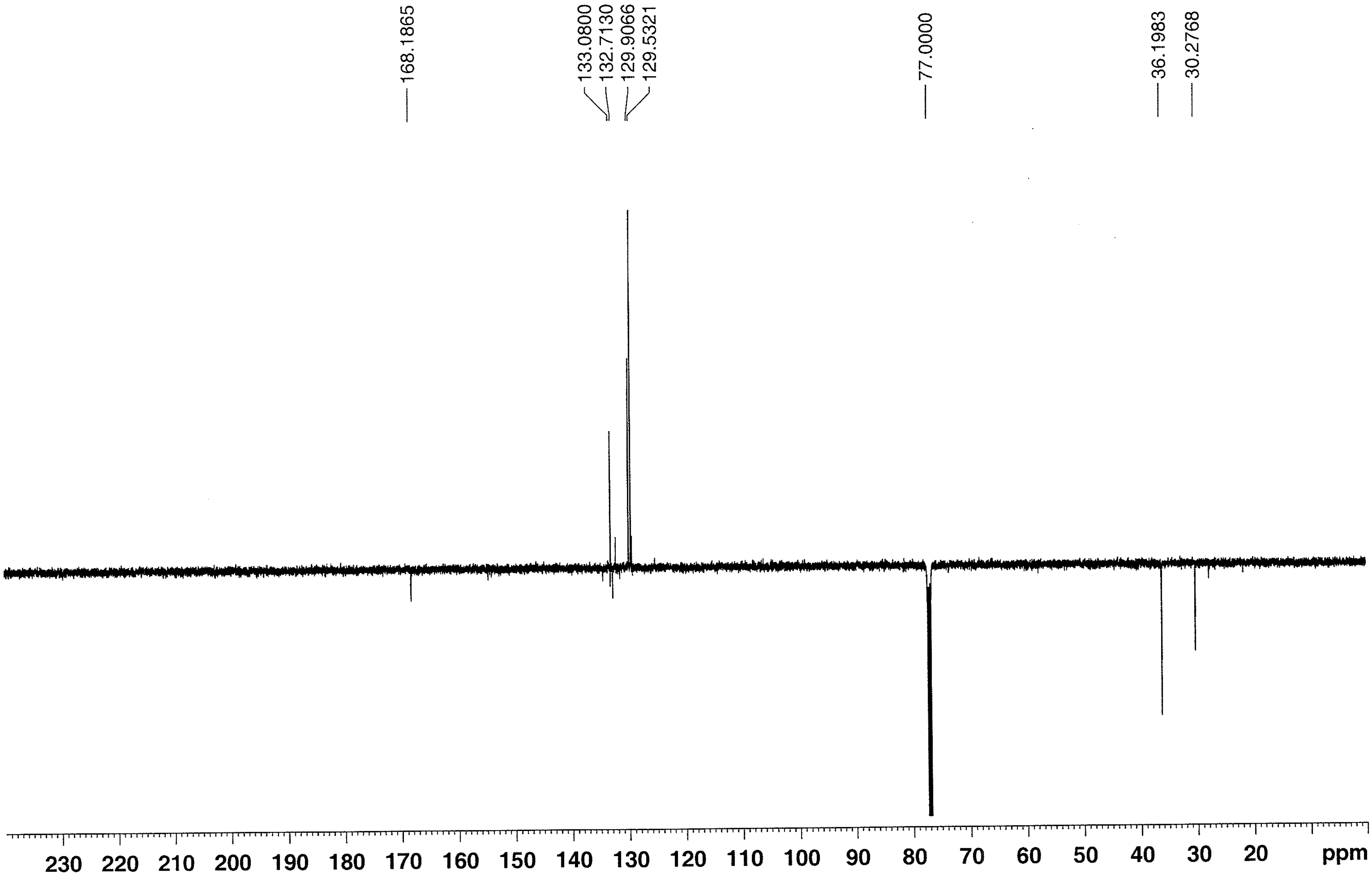

AWEPOpd06 in cdcl3 (APT) 2.11.2016

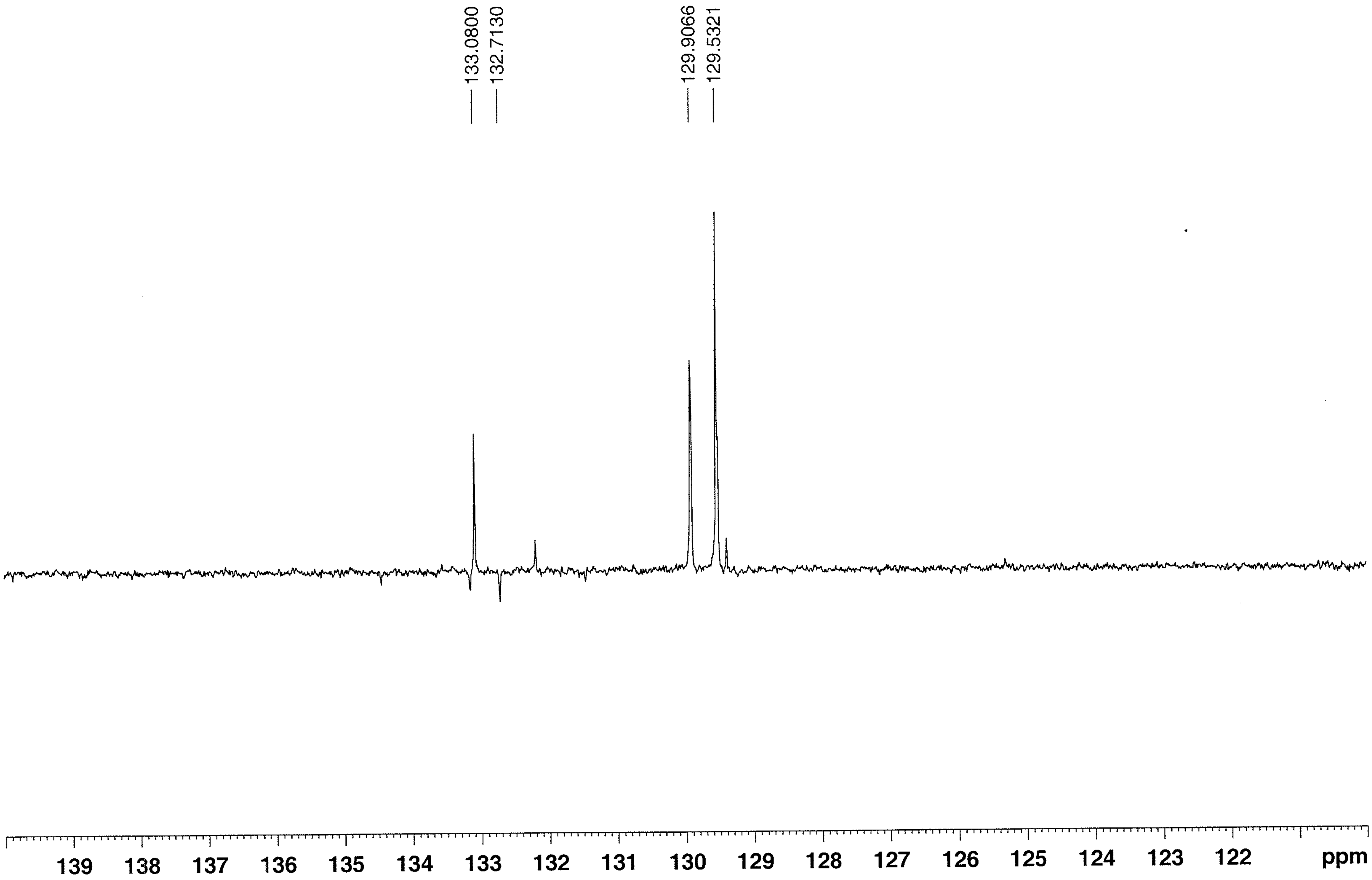

AWEPOpd06 in cdcl3 (APT) 2.11.2016

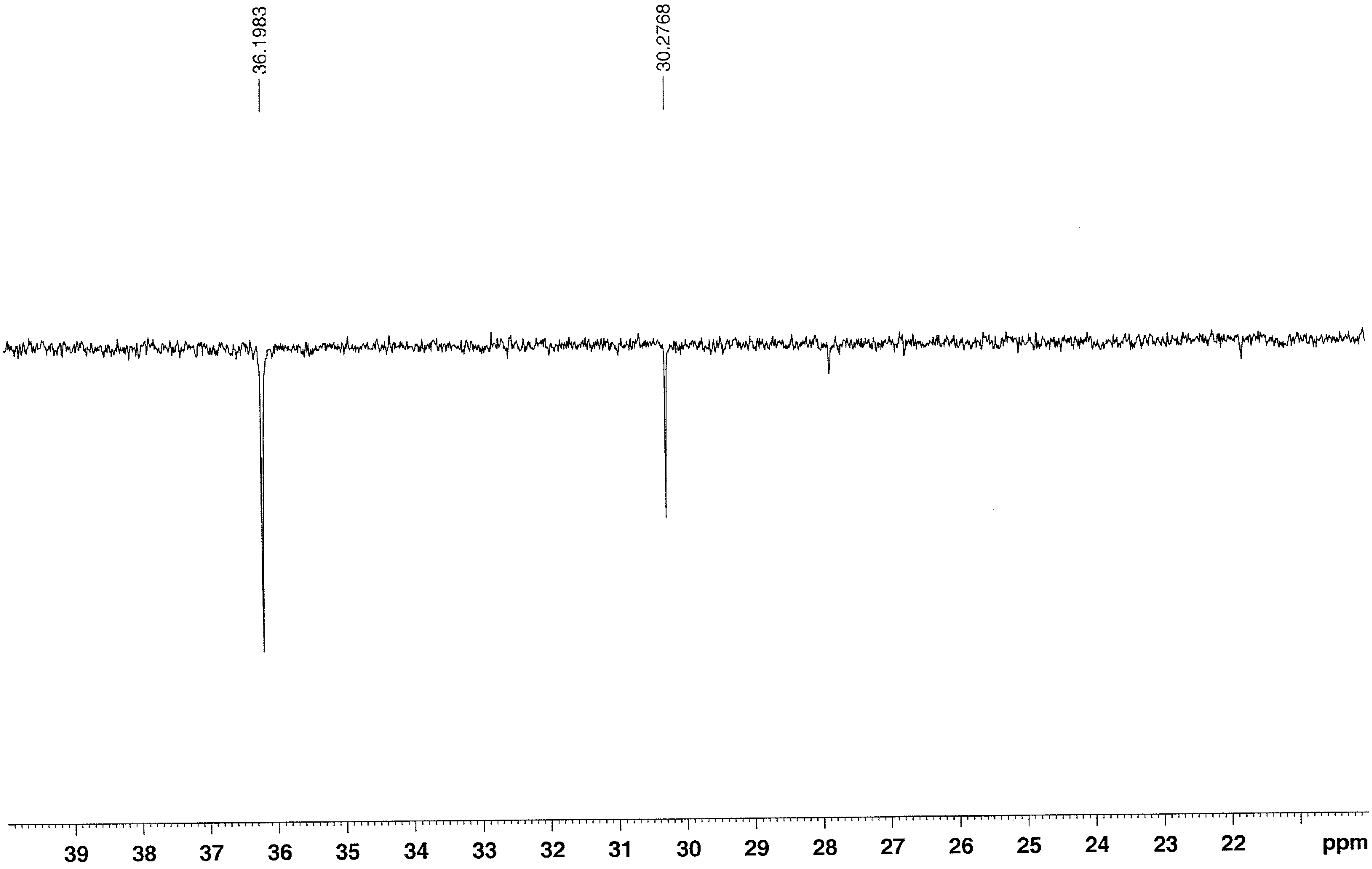

Supplement: Supplemental Material [file IENZ_A_1512598_SM8200.zip › IENZ_1512598_Supplementary Materials/Supplemental Information B_marked.pdf]
